# Novel meriolin derivatives activate the mitochondrial apoptosis pathway in the presence of antiapoptotic Bcl-2

**DOI:** 10.1038/s41420-024-01901-y

**Published:** 2024-03-09

**Authors:** Laura Schmitt, Ilka Lechtenberg, Daniel Drießen, Hector Flores-Romero, Margaretha A. Skowron, Marlena Sekeres, Julia Hoppe, Karina S. Krings, Tanya R. Llewellyn, Christoph Peter, Björn Stork, Nan Qin, Sanil Bhatia, Daniel Nettersheim, Gerhard Fritz, Ana J. García-Sáez, Thomas J. J. Müller, Sebastian Wesselborg

**Affiliations:** 1 Institute for Molecular Medicine I, Medical Faculty and University Hospital Düsseldorf, Heinrich Heine University Düsseldorf, Universitätsstraße 1, 40225 Düsseldorf, Germany; 2 Institute of Organic Chemistry and Macromolecular Chemistry, Faculty of Mathematics and Natural Sciences, Heinrich Heine University Düsseldorf, Universitätsstraße 1, 40225 Düsseldorf, Germany; 3 Institute for Genetics, Faculty of Mathematics and Natural Sciences, University of Cologne, 50931 Cologne, Germany; 4 Cologne Excellence Cluster on Cellular Stress Responses in Aging-Associated Diseases (CECAD), University of Cologne, 50931 Cologne, Germany; 5 Ikerbasque, Basque Foundation for Science, 48013 Bilbao, Spain; 6 Achucarro Basque Center for Neuroscience, Leioa, Spain; 7 Department of Urology, Urological Research Laboratory, Translational UroOncology, Medical Faculty and University Hospital Düsseldorf, Heinrich Heine University Düsseldorf, Moorenstraße 5, Düsseldorf, Germany; 8 Institute of Toxicology, Medical Faculty and University Hospital Düsseldorf, Heinrich Heine University Düsseldorf, Universitätsstraße 1, 40225 Düsseldorf, Germany; 9 Clinic of Hematology, Oncology and Clinical Immunology, Medical Faculty and University Hospital Düsseldorf, Heinrich Heine University Düsseldorf, Moorenstraße 5, 40225 Düsseldorf, Germany; 10 Department of Pediatric Oncology, Hematology and Clinical Immunology, Medical Faculty and University Hospital Düsseldorf, Heinrich Heine University Düsseldorf, Moorenstraße 5, 40225 Düsseldorf, Germany

**Keywords:** Drug development, Pharmaceutics, Apoptosis

## Abstract

Meriolin derivatives represent a new class of kinase inhibitors with a pronounced cytotoxic potential. Here, we investigated a newly synthesized meriolin derivative (termed meriolin 16) that displayed a strong apoptotic potential in Jurkat leukemia and Ramos lymphoma cells. Meriolin 16 induced apoptosis in rapid kinetics (within 2–3 h) and more potently (IC_50_: 50 nM) than the previously described derivatives meriolin 31 and 36 [1]. Exposure of Ramos cells to meriolin 16, 31, or 36 for 5 min was sufficient to trigger severe and irreversible cytotoxicity. Apoptosis induction by all three meriolin derivatives was independent of death receptor signaling but required caspase-9 and Apaf-1 as central mediators of the mitochondrial death pathway. Meriolin-induced mitochondrial toxicity was demonstrated by disruption of the mitochondrial membrane potential (ΔΨm), mitochondrial release of proapoptotic Smac, processing of the dynamin-like GTPase OPA1, and subsequent fragmentation of mitochondria. Remarkably, all meriolin derivatives were able to activate the mitochondrial death pathway in Jurkat cells, even in the presence of the antiapoptotic Bcl-2 protein. In addition, meriolins were capable of inducing cell death in imatinib-resistant K562 and KCL22 chronic myeloid leukemia cells as well as in cisplatin-resistant J82 urothelial carcinoma and 2102EP germ cell tumor cells. Given the frequent inactivation of the mitochondrial apoptosis pathway by tumor cells, such as through overexpression of antiapoptotic Bcl-2, meriolin derivatives emerge as promising therapeutic agents for overcoming treatment resistance.

## Introduction

Multikinase inhibitors interfere with cell proliferation, apoptosis, inflammation, and metabolism and are, therefore, successfully applied in cancer therapy [2–5]. So far, 66 protein kinase inhibitors have been approved by the FDA that are predominantly applied in cancer treatment [6].

**Fig. 1:**
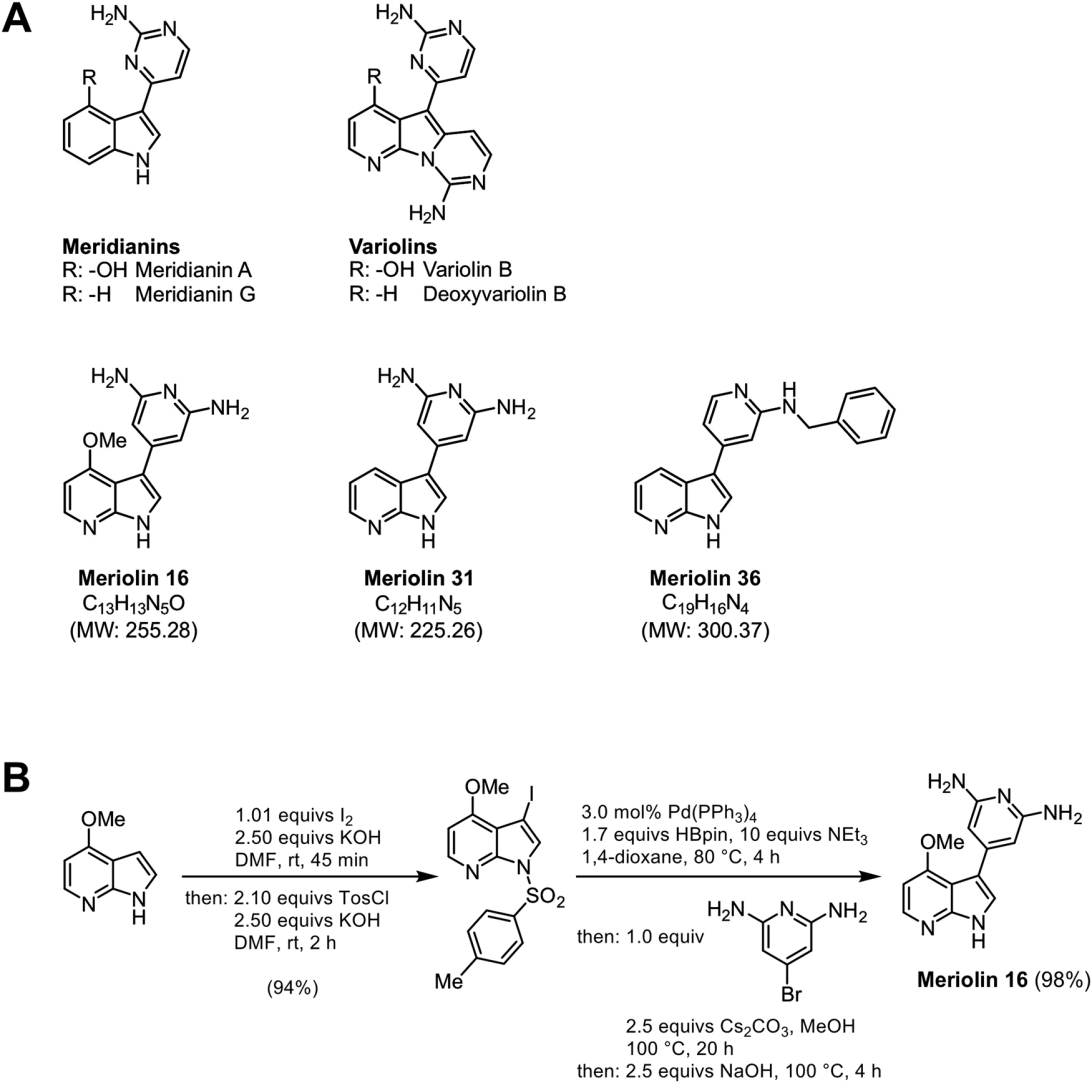
Structure of the natural products of meridianins, variolins, and their structural hybrids meriolin 16, 31, and 36. **A** Meridianins originate from the Ascidian *Aplidium meridianum* [68], whereas variolins were initially extracted from the Antarctic sponge *Kirkpatrickia variolosa* [69, 70]. The respective synthesis pathways of meriolin 31 and 36 have been previously described [1]. Meriolin 16 is a newly synthesized derivative of meriolin 31. **B** Synthesis of meriolin 16 from 4-methoxy 7-azaindole by iodination-tosylation sequence followed by Masuda borylation-Suzuki coupling sequence.

Meriolins (3-(pyrimidin-4-yl)-7-azaindoles) are synthetic hybrids of the naturally occurring (aza)indole alkaloid family of variolins and meridianins and exhibit potent kinase inhibitory features [7, 8] (Fig. 1A). They were first synthesized in 2001 [9] and termed meriolins by Meijer and co-workers [7, 10]. Similar to their parental natural products, meriolins inhibit a broad range of cyclin-dependent kinases (CDKs) and are even more potent than variolins and meridianins in vitro and in vivo [1, 7, 10–14]. Previous studies could show that meriolins act as multikinase inhibitors by inhibiting a variety of kinases such as CDKs (CDK1, 2, 4, 5, and 9) [7, 10, 15, 16] and to a lesser extent other kinases such as GSK-3, CK1, DYRK1A [10], sphingosine kinase 1 and 2 [1, 17], and PDK1 [18]. It has been shown that meriolins display a distinct cytotoxicity against several cancer cell lines with IC_50_ values in the nanomolar range [1, 7, 10, 12, 13]. In addition, it was demonstrated that meriolins induce cell cycle arrest [11, 19] and apoptosis [1, 7, 10, 11, 20] and consequently display antitumor activity in different xenograft cancer models (i.e., Ewing’s sarcoma, LS-174T colorectal carcinoma, and U87 glioblastoma) [7, 11]. Since dysregulation of cell cycle and aberrant proliferation represent key hallmarks of cancer [21, 22], targeting of CDKs represents a promising approach for anticancer therapy [4, 5]. In this context, meriolin 3 (that inhibits CDK1, CDK2, CDK5, and CDK9) has found its way into preclinical trials [16].

We have previously shown that out of 13 newly synthesized meriolin derivatives, 6 displayed a pronounced cytotoxicity in Jurkat T cell leukemia and Ramos Burkitt B cell lymphoma cells. Among these tested meriolin derivatives, meriolin 31 and 36 (Fig. 1A) showed the highest cytotoxic potential [1].

Here, we characterize the apoptosis signaling of the novel derivative meriolin 16 in comparison to the potent derivatives meriolin 31 and 36. Meriolin 16 was developed and synthesized with an additional methoxy group (Fig. 1A, B) in the hope to enhance its potential and accordingly displayed a higher cytotoxicity (IC_50_: 50 nM) than meriolin 31 and 36 (IC_50_: 90 nM and 170 nM) and even as meriolin 3 (IC_50_: 70 nM), which is the most potent meriolin derivative described so far. Interestingly, a 5 min exposure to meriolin 16, 31, or 36 was sufficient to activate the endogenous suicide program upon 24 h incubation. Meriolin-induced apoptosis was independent of external death receptor signaling but relied on Apaf-1 and caspase-9 as central mediators of the mitochondrial apoptosis pathway. Activation of the intrinsic mitochondrial death pathway was further confirmed by the breakdown of the mitochondrial membrane potential (ΔΨm), mitochondrial release of proapoptotic Smac, processing of the dynamin-like GTPase OPA1 and subsequent mitochondrial fission. Intriguingly, all three meriolin derivatives could activate the mitochondrial apoptosis pathway in Jurkat cells even in the presence of antiapoptotic Bcl-2 protein. Since tumor cells frequently inactivate the mitochondrial death pathway in order to acquire therapy resistance (e.g., by elevated levels of antiapoptotic Bcl-2), these meriolin derivatives display a favorable option for overcoming treatment resilience. In this context, we could show that meriolins were able to induce cell death in imatinib-resistant K562 and KCL22 chronic myeloid leukemia cells and in cisplatin-resistant J82 urothelial carcinoma and 2102EP germ cell tumor cells. Thus, the tested meriolin derivatives represent a promising therapeutic option for cancer treatment.

## Results

### Synthesis of meriolin 16 by Masuda borylation-Suzuki coupling sequence

Starting from 4-methoxy 7-azaindole, meriolin 16 was prepared in concise two-step synthesis (Fig. 1B). First, 4-methoxy 7-azaindole was iodinated and *N*-tosylated in a one-pot fashion according to our literature protocol [23] to give 3-iodo-4-methoxy-1-tosyl-1H-pyrrolo[2,3-b]pyridine in 94% yield. Then, the Masuda borylation-Suzuki coupling sequence [8] of 3-iodo-4-methoxy-1-tosyl-1H-pyrrolo[2,3-b]pyridine and 4-bromopyridine-2,6-diamine followed by subsequent detosylation yielded meriolin 16 in 98% after three steps in a one-pot fashion [24].

### Meriolin 16, 31, and 36 are highly cytotoxic and induce apoptosis in rapid kinetics in Ramos and Jurkat cells

**Fig. 2:**
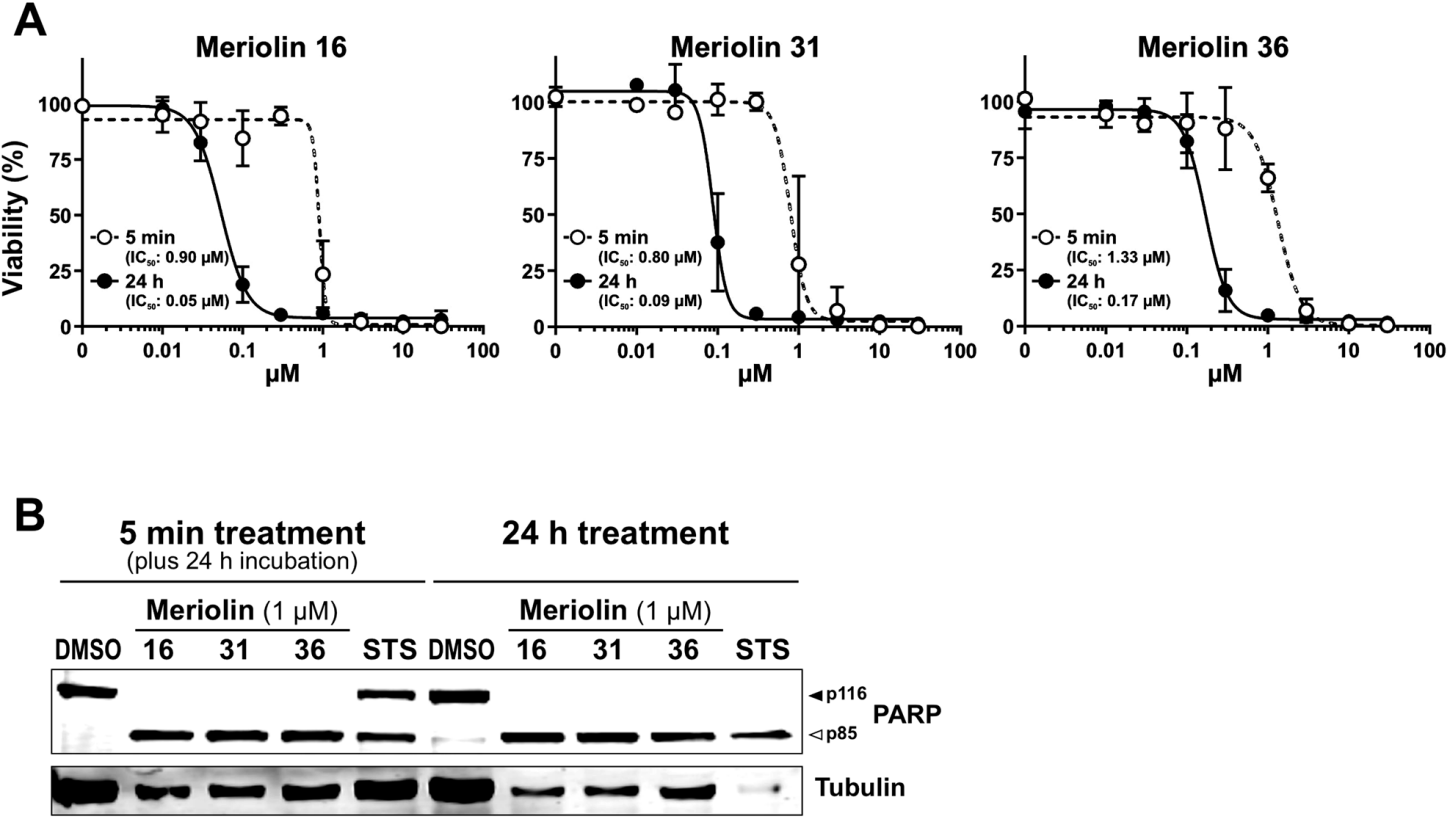
The Meriolin derivatives 16, 31, and 36 trigger irreversible cell death in Ramos lymphoma cells. **A** About 5 × 10^5^ Ramos Burkitt B cell lymphoma cells were treated with increasing concentrations of meriolin 16, 31, and 36 for 5 min, or 24 h. In the first setting, meriolins were washed away after 5 min and cells were further incubated in a medium. After 24 h upon start of the experiment, viability was determined by AlamarBlue^®^ assay. The respective IC_50_ values are given in parenthesis. **B** Ramos cells were treated with 0.1% (v/v) DMSO (diluent control), 1 µM of meriolin 16, 31, and 36 or 2.5 µM of staurosporine (STS; as a positive control for apoptosis-induction) for 5 min and the stimulus was removed after treatment. Subsequently, cleavage of the caspase-3 substrate poly(ADP-ribose) polymerase-1 (PARP), as an indicator for apoptotic cell death, was determined after 24 h of incubation via immunoblotting in comparison to 24 h without stimulus removal. Solid arrowheads indicate the uncleaved form of PARP (p116); open arrowheads indicate the cleaved form (p85). Immunoblotting for tubulin was used as loading control.

To evaluate the cytotoxic potential of the novel derivative meriolin 16 in comparison to meriolin 31 and 36, we performed viability assays. All three meriolin derivatives were extremely cytotoxic in Ramos lymphoma cells with IC_50_ values ranging from 50 nM (meriolin 16) and 90 nM (meriolin 31) to 170 nM (meriolin 36) upon 24 h treatment (Fig. 2A). Thus, the IC_50_ value of meriolin 16 was even lower than for meriolin 3 (IC_50_: 70 nM; Suppl. Fig. 1A), which is the most potent meriolin derivative described so far. In addition, we treated Ramos cells for only 5 min with meriolin 16, 31, or 36. Subsequently, cells were washed and further incubated for 24 h. Thereby, we observed that the exposure to the respective meriolin derivative for 5 min was sufficient to cause severe and irreversible cytotoxicity (Fig. 2A). Similarly, a 5 min exposure to meriolins was sufficient for the cleavage of the caspase substrate poly(ADP-ribose) polymerase-1 (PARP) after 24 h. Interestingly, PARP-cleavage upon 5 min exposure to all three meriolin derivatives was more pronounced than 5 min treatment with the broad kinase inhibitor and potent apoptotic stimulus staurosporine (STS), which was used as a positive control (Fig. 2B).

**Fig. 3:**
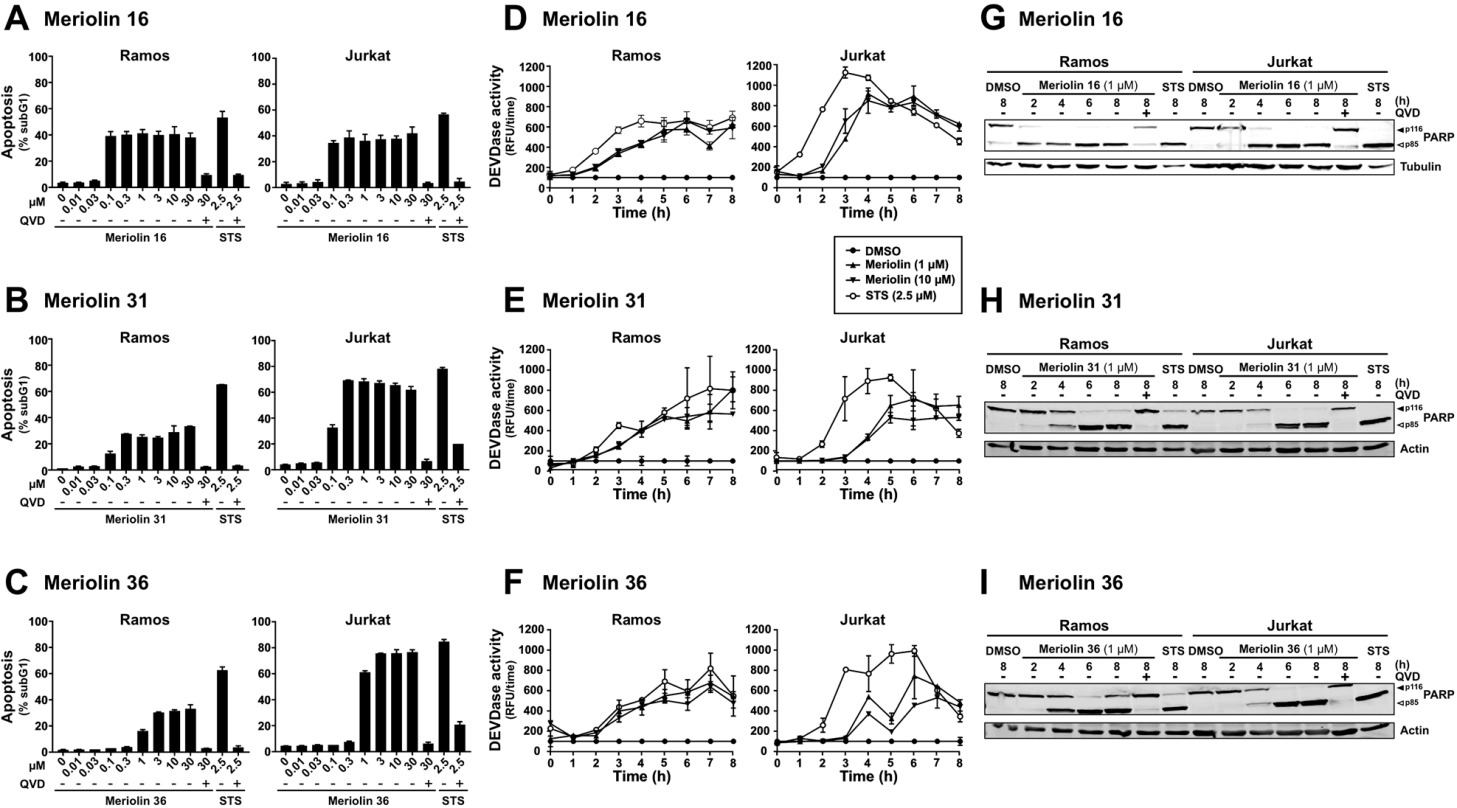
Meriolin 16, 31, and 36 induce apoptosis in rapid kinetics in leukemia and lymphoma cells. **A**–**C** About 5 × 10^5^ Ramos lymphoma or Jurkat leukemia cells were treated with increasing concentrations of meriolin 16 (**A**), 31 (**B**), and 36 (**C**) or 2.5 µM staurosporine (STS), either alone or in combination (pre- and cotreatment) with the pan-caspase inhibitor QVD (10 µM). After 24 h of incubation, apoptosis-related DNA degradation was detected via flow-cytometric measurement of propidium iodide-stained apoptotic hypodiploid nuclei [25]. Error bars = SD of triplicates. **D**–**F** About 5 × 10^5^ Ramos or Jurkat cells were treated with 1 or 10 µM of meriolin 16 (**D**), 31 (**E**), and 36 (**F**) or 2.5 µM staurosporine (STS) for up to 8 h. Subsequently, caspase-3 activity was determined by measurement of the fluorescence of the profluorescent caspase-3 substrate DEVD-AMC in a spectrofluorometer. Error bars = SD of triplicates. **G**–**I** About 1 × 10^6^ Ramos or Jurkat cells were treated with 1 µM of meriolin 16 (**G**), 31 (**H**), and 36 (**I**), 0.1% (v/v) DMSO (solvent control), or 2.5 µM staurosporine (STS) for the indicated time periods, either alone or in combination (pre- and cotreatment) with the pan-caspase inhibitor QVD (10 µM). Cleavage of the caspase substrate PARP was detected by immunoblotting. Solid arrowheads indicate the uncleaved form of PARP (p116); open arrowheads indicate the cleaved form (p85). Immunoblotting for tubulin or β-actin was used as a loading control.

To further evaluate the apoptotic potential, we monitored the apoptosis-related DNA degradation by the flow-cytometric measurement of propidium iodide-stained apoptotic hypodiploid nuclei [25]. Again, meriolin 16 turned out to be more potent and to induce apoptosis at lower concentrations compared to meriolin 31 and 36 and to a similar extent in both, Jurkat leukemia and Ramos lymphoma cells. Meriolin-induced apoptosis was prevented by cotreatment with the pan-caspase inhibitor Q-VD-OPh (QVD), thereby indicating that cell death was caspase-dependent (Fig. 3A–C). To monitor the catalytic caspase activity, we used the profluorescent caspase-3 substrate Ac-DEVD-AMC. The measurement of 8-h kinetics revealed a rapid activation of caspase-3 as early as 3–4 h in Ramos and Jurkat cells upon treatment with different meriolin derivatives. However, meriolin-induced caspase-activation was not quite as rapid as upon treatment with staurosporine (Fig. 3D–F). Moreover, a significant cleavage of the caspase substrate PARP was observed in both cell lines, displaying kinetics analogous to those detected in the caspase activity assay, which was prevented by cotreatment with the pan-caspase inhibitor QVD (Fig. 3G–I).

### Meriolins induce DNA damage which is a downstream event mediated by caspases

It has been demonstrated that CDK1 phosphorylates and activates the dynamin-related GTPase DRP1 during mitosis [26]. Conversely, loss of the fission protein DRP1 causes mitochondrial hyperfusion and induces ATM-dependent G2/M arrest and aneuploidy through DNA replication stress [27]. Therefore, we investigated whether the meriolin-induced CDK1 inhibition might trigger DNA damage, and subsequently initiate the DNA damage response. For this purpose, apoptosis induction upon treatment with meriolin 16 and 36, as well as the CDK1, 2, and 4 inhibitor R547 [28], was compared with the topoisomerase inhibitors and DNA damaging agents camptothecin, daunorubicin, and etoposide. As shown in Suppl. Fig. 2A, all tested apoptosis stimuli induced pronounced caspase activity in a similar range and kinetics. Next, we investigated the effect of CDK-inhibition by meriolins and R547 on the phosphorylation of the two checkpoint kinases CHK1 and CHK2. Both are responsible for cell cycle arrest upon DNA damage or during incomplete cell cycle progression. Upon DNA damage, CHK1 and CHK2 become phosphorylated by ATM or ATR [29]. ATR is activated by DNA single-strand breaks and other damage during the replication process and subsequently phosphorylates CHK1 at Ser345. CHK2 is phosphorylated at Thr68 by ATM, which is activated by DNA double-strand breaks. As depicted in Suppl. Fig. 2B, C, all three CDK-inhibitors (meriolin 16, 36, and R547) induced a pronounced phosphorylation of CHK2, indicating the induction of DNA double-strand breaks, whereas CHK1 remained merely unaffected. Another biomarker for DNA double-strand breaks is the phosphorylation of γH2AX at Ser139 by ATM as a first step in the recruitment of DNA repair proteins [30]. In addition to γH2AX, ATM also phosphorylates KAP1 at Ser824 [31], which subsequently co-localizes with γH2AX, TP53BP1, and BRCA1 at DNA damage foci [32]. Treatment of Ramos cells with meriolin 16 induced a substantial phosphorylation of γH2AX (at Ser139) and KAP1 (at Ser824). However, pretreatment with the pan-caspase inhibitor QVD completely abolished meriolin 16 induced phosphorylation of γH2AX and KAP1, whereas etoposide-mediated phosphorylation was not affected (Suppl. Fig. 2D, E). Accordingly, ATM-mediated phosphorylation of CHK2 at Thr68 was also abrogated in the presence of QVD (Suppl. Fig. 3). Thus, these data indicate that in contrast to etoposide, which inflicts DNA damage in a direct way, meriolin 16 induced DNA damage is rather an apoptotic downstream event mediated by caspases.

### Meriolin 16, 31, and 36 activate the mitochondrial apoptosis pathway even in the presence of antiapoptotic Bcl-2

Next, we investigated whether the meriolin derivatives affected the different apoptotic signaling pathways. There are at least two major pathways governing the apoptotic demise of a cell—the extrinsic death receptor pathway and the intrinsic mitochondrial apoptosis pathway. Death receptor-mediated apoptosis is initiated by stimulation of receptors (such as CD95/Apo-1/Fas, TRAIL-R1, or TRAIL-R2) by their respective ligands (e.g., CD95L/Apo-1L/FasL or TRAIL). This, in turn, activates initiator caspase-8, which subsequently activates the effector caspase-3.

The mitochondrial death pathway is triggered by cellular stress, such as DNA damage induced during radio- and chemotherapy. It is initiated by the mitochondrial release of cytochrome c, which is mediated by proapoptotic Bcl-2 proteins, such as Bax or Bak. Antiapoptotic Bcl-2 proteins (e.g., Bcl-2, Bcl-xL, Mcl-1) inhibit the mitochondrial cytochrome c release by counteracting proapoptotic Bcl-2 members and thereby impeding the mitochondrial death pathway [33]. Within the cytosol, cytochrome c associates with the adapter protein Apaf-1 which induces the activation of initiator caspase-9 in a high molecular weight signal complex, termed apoptosome. Once activated, caspase-9 proteolytically activates effector caspase-3 and -7 [34, 35].

**Fig. 4:**
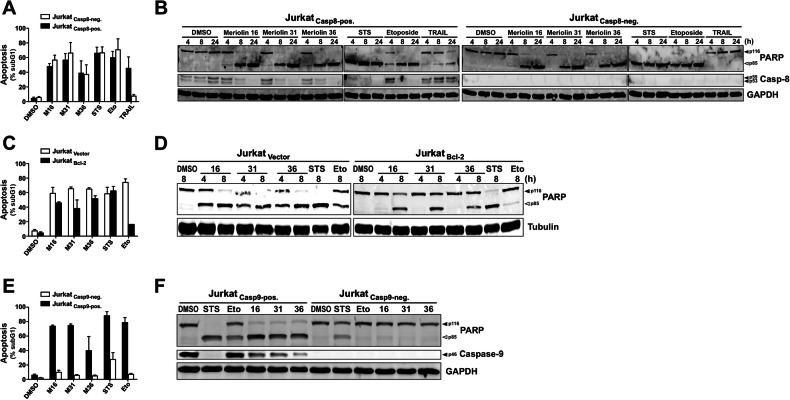
Meriolin 16, 31, and 36 activate the mitochondrial apoptosis pathway in Bcl-2 overexpressing Jurkat cells. **A**, **B** Meriolins do not induce apoptosis via the death receptor pathway. **A** About 5 × 10^5^ caspase-8 proficient Jurkat cells (Jurkat-Casp8-pos., black bars) or caspase-8 deficient Jurkat cells (Jurkat-Casp8-neg., white bars) [36] were treated with 0.1% (v/v) DMSO (solvent control), 1 µM of meriolin 16, 31, and 36, 50 µM of etoposide (Eto), 2.5 µM staurosporine (STS), or the death receptor ligand TRAIL (40 ng/ml; positive control). After 24 h, apoptosis was assessed by propidium iodide staining of apoptotic hypodiploid nuclei and flow cytometry. **B** About 1 × 10^6^ caspase-8 proficient Jurkat cells (Jurkat-Casp8-pos.) or caspase-8 deficient Jurkat cells (Jurkat-Casp8-neg.) were treated as in (A) for the indicated time period. Cleavage of the caspase substrate PARP was detected by immunoblotting. Solid arrowheads indicate the uncleaved form of PARP (p116); open arrowheads indicate the cleaved form (p85). **C**, **D** Meriolin 16, 31, and 36 induce apoptosis in the presence of antiapoptotic Bcl-2. Jurkat cells stably transfected with vectors encoding Bcl-2 (Jurkat Bcl-2; black bars) [60] or empty vector (Jurkat vector; white bars) were treated with 0.1% (v/v) DMSO, 1 µM of meriolin 16, 31, and 36, 50 µM etoposide (Eto), or 2.5 µM staurosporine (STS). **C** After 24 h, apoptosis was assessed by flow-cytometric measurement of apoptotic hypodiploid nuclei. **D** After the indicated time period, cleavage of the caspase substrate PARP was detected by immunoblotting. **E**, **F** Meriolin-induced apoptosis requires caspase-9. Caspase-9 deficient (Jurkat Casp9-neg., white bars) or caspase-9 proficient Jurkat cells (Jurkat Casp9-pos., black bars) [59] were treated with 0.1% (v/v) DMSO, 1 µM of meriolin 16, 31, and 36, 50 µM etoposide (Eto), or 2.5 µM staurosporine (STS). **E** After 24 h, apoptosis was assessed by flow-cytometric measurement of apoptotic hypodiploid nuclei. **F** After the indicated time period, cleavage of the caspase substrate PARP was detected by immunoblotting. Immunoblot of expression of caspase-9 is also shown. **B**, **D**, **F** Immunoblotting for GAPDH or tubulin was used as loading control.

Given that caspase-8 serves as the primary initiator caspase in the death receptor pathway, we employed caspase-8 deficient Jurkat cells [36] to examine whether meriolin-triggered apoptosis operates through the extrinsic apoptosis pathway. As depicted in Fig. 4A, B, all three meriolin derivatives instigated apoptosis and cleavage of the caspase substrate PARP in both, parental caspase-8 proficient as well as in caspase-8 deficient Jurkat cells. Likewise, staurosporine and the anticancer drug etoposide, known for their capacity to induce apoptosis independently of external death receptor signaling [37, 38], were also effective in inducing apoptosis in caspase-8 deficient cells. As expected, in the absence of caspase-8 death receptor stimulation through TRAIL was consequently impeded. Thus, involvement of the external death receptor pathway in meriolin-induced apoptosis could be excluded.

Overexpression of antiapoptotic Bcl-2 proteins, including Bcl-2, Bcl-xL, or Mcl-1, has the capability to impede the activation of the mitochondrial apoptosis pathway. Intriguingly, in Jurkat cells overexpressing Bcl-2, the induction of apoptosis and PARP-cleavage by meriolin 16, 31, and 36 were only mitigated but not entirely obstructed (Fig. 4C, D and Suppl. Fig. 4A). However, the kinetics of PARP-cleavage (Fig. 4D) and apoptosis induction was delayed in Jurkat cells overexpressing Bcl-2 compared to vector control cells (Suppl. Fig. 4A). As anticipated, the initiation of apoptosis by the DNA-damaging anticancer drug etoposide was prevented in the presence of Bcl-2. Since staurosporine is able to induce apoptosis in Bcl-2 or Bcl-xL overexpressing cells [37], apoptosis induction was not affected by Bcl-2 (Fig. 4C, D and Suppl. Fig. 4A). However, when we used Jurkat cells overexpressing antiapoptotic Bcl-xL, meriolin-induced apoptosis was almost completely blocked (Suppl. Fig. 4B). Similarly, meriolin 16, 31 and 36 could also not induce apoptosis in the Bax- and Bak-deficient human B cell Burkitt lymphoma cell line DG75 (Suppl. Fig. 4C).

Given that the activation of the mitochondrial apoptosis pathway relies on the Apaf-1-mediated activation of caspase-9 within the apoptosome, we examined whether meriolins could initiate apoptosis in Jurkat cells with stable transcriptional repression of Apaf-1 achieved through CRISPR interference. In addition, we used the melanoma cell line SK-Mel-94 which had been previously demonstrated to be resistant to anticancer drugs due to the absence of Apaf-1 expression [39]. Thus, meriolin 16, 31, and 36 mediated apoptosis induction was substantially reduced in Apaf-1-knockdown Jurkat cells (Suppl. Fig. 4D) or caspase-activation in Apaf-1 deficient SK-Mel-94 cells (Suppl. Fig. 4E). Since anticancer drugs induce apoptosis via the mitochondrial apoptosis pathway, etoposide was also unable to induce apoptosis in Apaf-1 deficient Jurkat cells and SK-Mel-94 cells. As staurosporine is capable to activate caspase-9 in the absence of Apaf-1 [37], apoptosis induction and caspase-activation was not obstructed (Suppl. Fig. 4D, E). The observation that meriolin 16, 31, and 36 obviously required apoptosome formation was further supported by the observation that meriolin-induced apoptosis and PARP-cleavage was completely abrogated in caspase-9 deficient Jurkat cells (Fig. 4E, F).

**Fig. 5:**
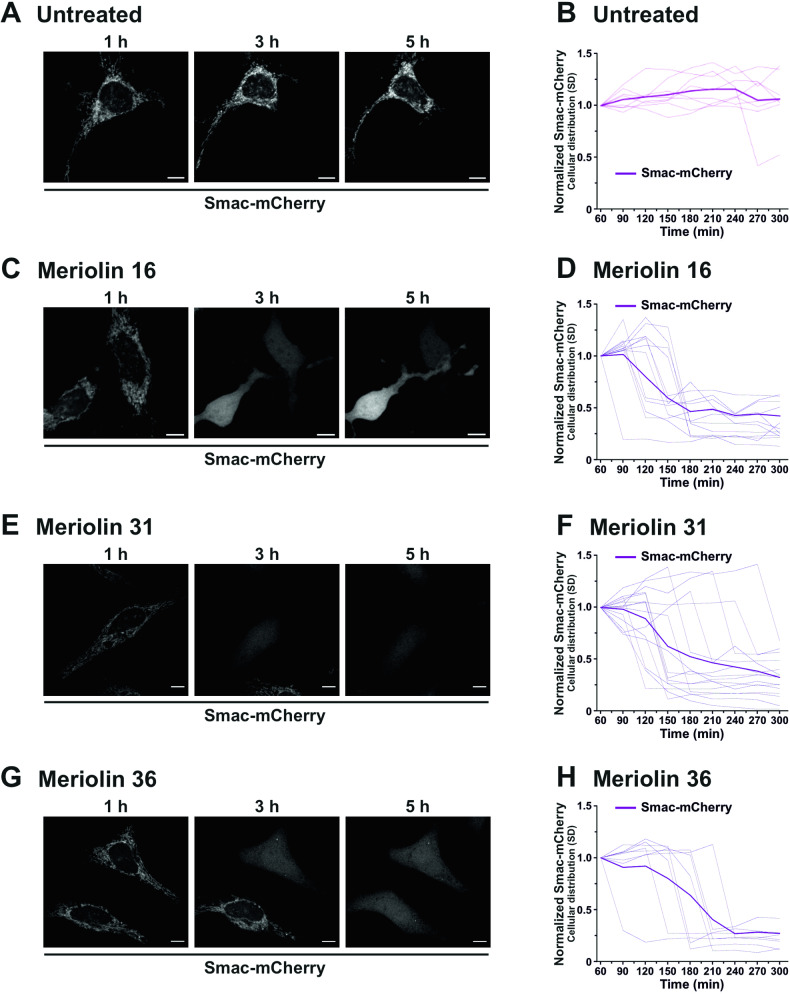
Meriolin 16, 31 and 36 induce mitochondrial release of the proapoptotic protein Smac. **A**–**H** Confocal live imaging of HeLa cells expressing Smac-mCherry after treatment with 0.1% v/v DMSO (Untreated) (**A**, **B**), 1 µM of meriolin 16 (**C**, **D**), 31 (**E**, **F**), or 36 (**G**, **H**) for the indicated time points. **A**, **C**, **E**, **G** Confocal imaging. Scale bars 5 µm. **B**, **D**, **F**, **H** Quantification of the effects of meriolins on mitochondrial Smac-mCherry release. The normalized standard deviation (SD) of the fluorescence intensity of Smac-mCherry upon treatment with 0.1% v/v DMSO (Untreated) or 1 µM of the indicated meriolins was used as an indicator of its subcellular distribution in individual cells (*N* = 8–15) from two independent experiments. A low SD corresponds to homogenous distribution (cytosolic), while a high SD is related to confined localization (mitochondria). Thinner lines represent measurements of individual cells, while thicker lines represent the average of all recorded cells.

Activation of the mitochondrial death pathway is initiated by the release of proapoptotic factors such as cytochrome c and Smac (also known as Diablo) from mitochondria [35]. To further corroborate the involvement of the mitochondrial apoptosis pathway, we monitored the mitochondrial release of Smac-mCherry in HeLa cells. Thus, we could show that within 2–3 h, all three meriolin derivatives initiated the release of Smac from mitochondria (Fig. 5). Taken together, these data indicate that all three meriolin derivatives can activate the mitochondrial apoptosis pathway that could not be blocked by Bcl-2 overexpression.

### Meriolin 16, 31, and 36 impair mitochondrial function and structure

**Fig. 6:**
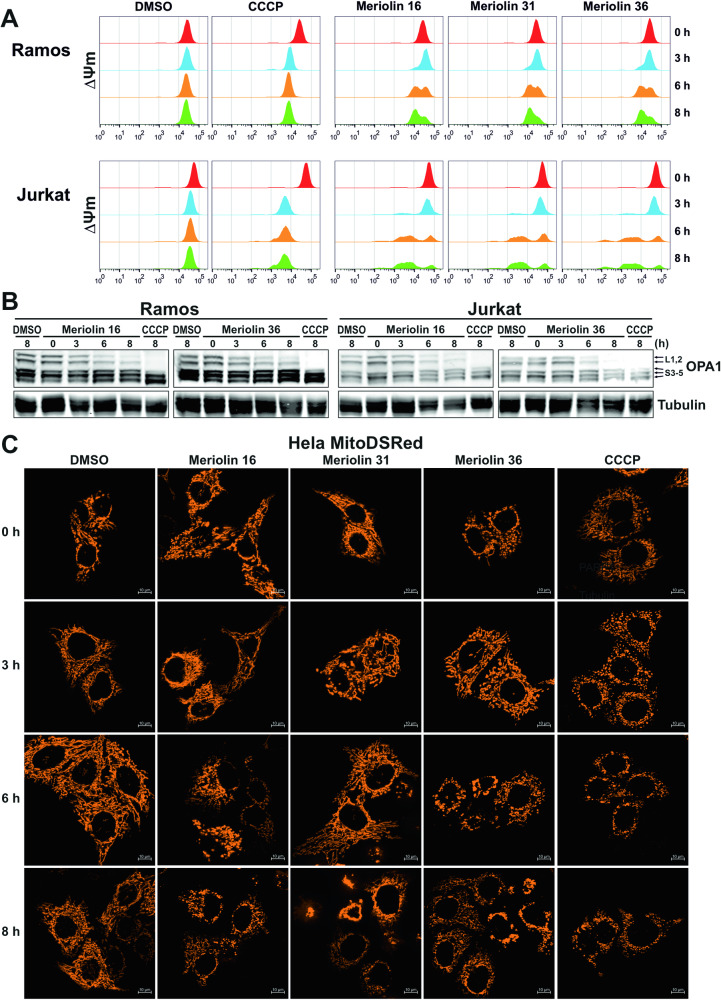
Meriolin 16, 31, and 36 impair mitochondrial structure and function. **A** Monitoring of the mitochondrial membrane potential (ΔΨm) of Ramos and Jurkat cells upon addition of 10 µM of meriolin 16, 31, or 36, 0.1% (v/v) DMSO (diluent control) or 10 µM CCCP (mitochondrial uncoupler, positive control) by flow-cytometric measurement of TMRE fluorescence. **B** The kinetics of the cleavage of the long isoforms (L1,2) of the dynamin-like GTPase OPA1 was determined by immunoblotting in Ramos (left panel) and Jurkat cells (right panel). Cells were treated as in (**A**) for the indicated time points. Immunoblotting for tubulin was used as a loading control. **C** Meriolin 16, 31, and 36 induce mitochondrial fragmentation (fission) in HeLa cells, stably expressing the fluorescent dye mito-DsRed targeted to the outer mitochondrial membrane. Cells were treated with DMSO (0.1% v/v), 1 µM of meriolin 16, 31, or 36, or 10 µM CCCP (positive control) for the indicated time points and mitochondrial morphology was assessed by microscopy (Apotome, Zeiss Axiovert). Shown are representative images.

Next, we focused on mitochondria as the executioners of the intrinsic apoptosis pathway. Therefore, we measured the effect of meriolin 16, 31, and 36 on the mitochondrial membrane potential (ΔΨm). As shown in Fig. 6A, all three meriolin derivatives mediated a significant disruption of the mitochondrial membrane potential (ΔΨm) after 6 h—however, not as fast as the protonophore carbonyl cyanide *m*-chlorophenyl hydrazone (CCCP), which was applied as a positive control.

In addition to the collapse of the mitochondrial membrane potential, the mitochondrial death pathway is characterized by mitochondrial fragmentation (fission). Mitochondrial fission is mediated in part by the proteolytic processing of the dynamin-like GTPase OPA1 by OMA1 and YME1L1, thereby balancing fusion and fission of the mitochondrial network. Thus, OPA1 plays as a crucial role in mitochondrial homeostasis by acting as a sensor of mitochondrial stress (e.g., ΔΨm breakdown) and integrating mitochondrial quality control and intrinsic apoptosis. Accordingly, stress-induced cleavage of OPA1 and the accompanied degradation of its long isoforms (L-OPA1) tilt the balance towards mitochondrial fragmentation and increased sensitivity to proapoptotic stimuli [40, 41]. Therefore, we analyzed the effect of meriolin 16 and 36 on OPA1 processing and could show that both meriolin derivatives induced the cleavage of the long OPA1 isoforms (L-OPA1) in Ramos and Jurkat cells within 6–8 h (Fig. 6B). We also noted that the loss of long OPA1 isoforms coincided with notable fragmentation (fission) of the mitochondrial network after 6–8 h (Fig. 6C).

### Meriolin 16 and 36 induce cytotoxicity in imatinib and cisplatin-resistant cancer cells

**Fig. 7:**
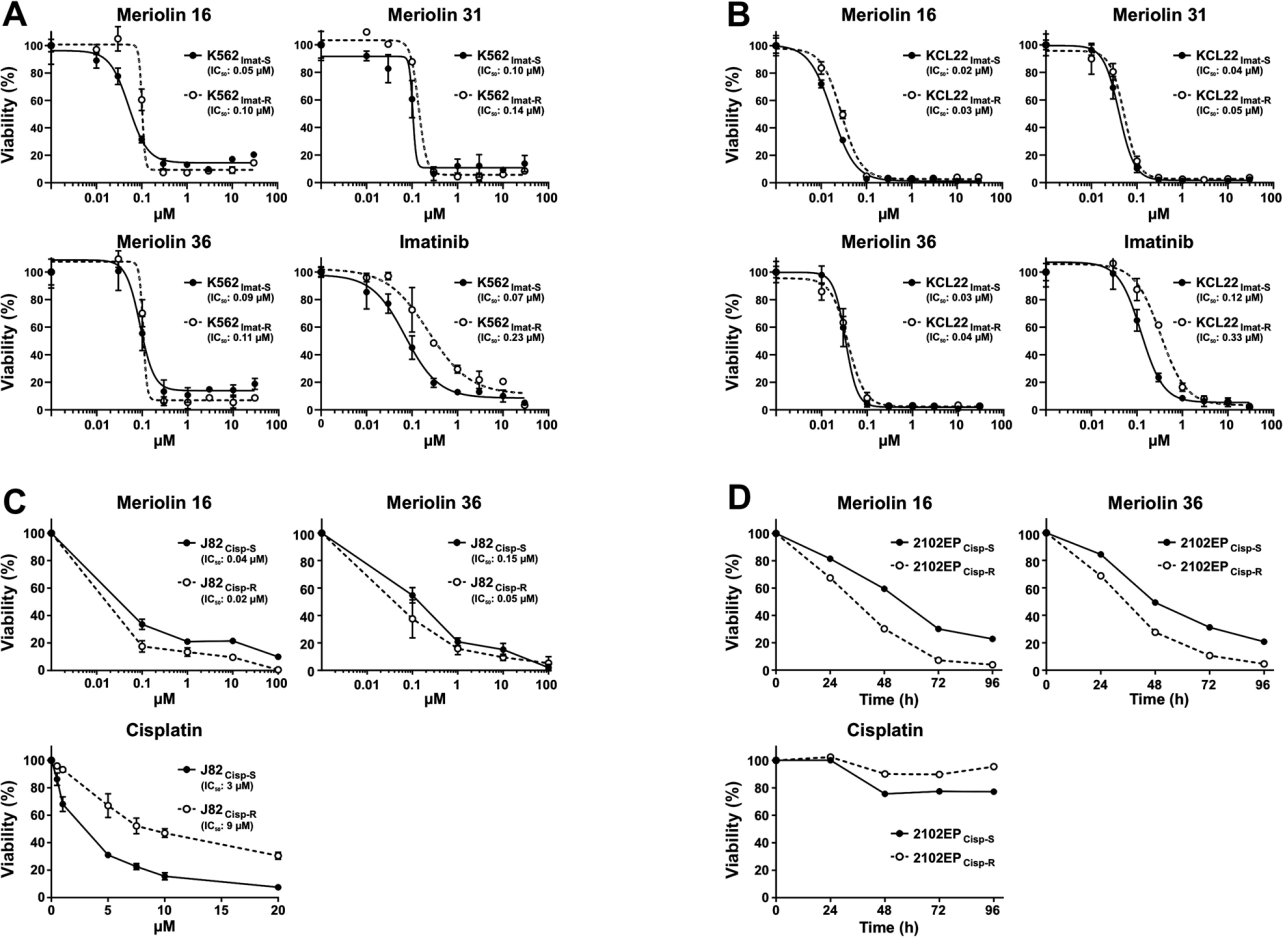
Meriolin 16, 31, and 36 induce cytotoxicity in imatinib- and cisplatin-resistant tumor cell lines. **A**, **B** Determination of cytotoxicity after 72 h treatment with meriolin 16 and 36 or imatinib in (**A**) imatinib-sensitive (K562 _Imat-S_) and -resistant (K562 _Imat-R_) K562 chronic myeloid leukemia (CML) cells or **B** imatinib-sensitive (KCL22 _Imat-S_) and -resistant (KCL22 _Imat-R_) KCL22 CML cells. Cell viability was assessed by AlamarBlue® assay. Respective IC_50_ values are shown in parenthesis. Data shown are the mean ± SD from one (*n* = 1) experiment, performed in triplicates (*n* = 3). **C** Determination of cytotoxicity after 72 h treatment with meriolin 16 and 36 or cisplatin in cisplatin-sensitive (J82_Cisp-S_) and -resistant (J82_Cisp-R_) J82 urothelial carcinoma cells. Cell viability was assessed by AlamarBlue® assay. Respective IC_50_ values are shown in parenthesis. Data shown are the mean ± SD from three independent (*n* = 3) experiments, each performed in quadruplicate (*n* = 4). **D** Kinetics of cytotoxicity upon treatment with 1 µM cisplatin, meriolin 16 or 36 of cisplatin-sensitive (2102EP_Cisp-S_) and -resistant (2102EP_Cisp-R_) 2102EP germ cell tumor cells. Cell viability was assessed by XTT assay.

The major detrimental feature of tumors is their potential to acquire resistance to radio- and chemotherapy. Consequently, preexisting or acquired resistance to anticancer drugs like imatinib or cisplatin treatment represents a major drawback that enables tumor progression. In order to evaluate the potential to overcome therapy resistance, we tested the meriolin derivatives in imatinib-resistant sublines of K562 and KCL22 chronic myeloid leukemia (CML) cells and cisplatin-resistant sublines of J82 urothelial cancer cells and 2102EP germ cell tumor cells in comparison to the respective imatinib- or cisplatin-sensitive parental cell lines [42–45]. As shown in Fig. 7, meriolin 16 and meriolin 36 displayed pronounced cytotoxicity after 72 h treatment in imatinib-resistant K562 and KCL22 cells as well as in cisplatin-resistant J82 and 2102EP cells and appeared to be even more cytotoxic compared to the respective sensitive parental cell lines.

## Discussion

As 7-azaindoles, meriolins represent structural hybrids of meridianins and variolins that have been isolated from marine invertebrates (Fig. 1A) [8, 46]. Similar to their parental natural counterparts, meriolins exhibit potent inhibition of a wide spectrum of CDKs and seem to demonstrate even greater activity compared to variolins and meridianins in vitro and in vivo [1, 8, 10–14]. Thus, it has been shown that meriolins display a strong cytotoxic potential in nanomolar range in various tumor cell lines of different origin, such as colon cancer (HCT116, LS-174T), hepatoma (Huh7, F1), cervix carcinoma (HeLa), breast carcinoma (MCF-7), glioma (GBM, SW1088, U87), neuroblastoma (SH-SY5Y), leukemia (Jurkat, Molt-4), lymphoma (Ramos), and myeloma (KMS-11) [1, 7, 11, 19, 20, 47]. However, non-transformed cells, such as human foreskin fibroblasts, appear to be more resilient [7]. In recent years, this substance class underwent derivatization and optimization in order to enhance its efficacy in cancer therapy. In this context, we have previously shown that 6 out of 13 newly synthesized meriolin derivatives displayed a pronounced cytotoxicity, with meriolin 31 and 36 comprising the highest cytotoxic potential [1].

Here, we investigated the bioactivity of the novel derivative meriolin 16 and compared its cytotoxic potential to meriolin 31 and 36. Thus, we could show that meriolin 16 was extremely cytotoxic (IC_50_: 50 nM) in Ramos lymphoma cells as compared to meriolin 31 (IC_50_: 90 nM) and meriolin 36 (IC_50_: 170 nM) upon 24 h treatment. Meriolin 16 was even more potent than meriolin 3 (IC_50_: 70 nM) that has already been tested in preclinical trials (Suppl. Fig. 1) [16]. Since meriolin 3 exhibits the highest cytotoxic potential amongst all meriolins described so far, this renders meriolin 16 as the most potent meriolin derivative to date.

The increased cytotoxicity of meriolin 16 compared to meriolin 31 and 36 might be attributed to the additional methoxy group at the aromatic pyridine ring of meriolin 16 (see Fig. 1A). The methoxy group was intentionally added in order to modify its pharmacokinetic properties and to enhance its biological activity. Besides affecting the polarity and solubility of a molecule, an additional methoxy group can increase potential reactivity by enhancing the electron density of the aromatic ring, thus rendering it more nucleophilic [48]. Since meriolin 3 also contains a methoxy group at the same position as meriolin 16 (Suppl. Fig. 1B), the methoxy group might be of importance for the structure-activity relationship of these derivatives.

Most interestingly, a 5-min exposure to meriolin 16, 31, or 36 was sufficient to activate the endogenous suicide program in Ramos lymphoma cells after 24 h (Fig. 2). We further investigated the apoptosis signaling pathways activated by meriolins. Using cell lines deficient for caspase-8, -9, or Apaf-1, we could show that meriolin-induced apoptosis was independent of external death receptor signaling but required Apaf-1 and caspase-9 as central mediators of the mitochondrial apoptosis pathway (Suppl. Fig. 4D, E and Fig. 4E, F). Activation of the mitochondrial apoptosis pathway was further confirmed by the mitochondrial release of proapoptotic Smac, breakdown of the mitochondrial membrane potential (ΔΨm), processing of the dynamin-like GTPase OPA1 and subsequent mitochondrial fission (Figs. 5, 6).

The question, however, remains, how meriolins activate the mitochondrial apoptosis pathway. We initially observed that meriolin 16 and 36 and other CDK-inhibitors, such as R547, induce the ATM-mediated phosphorylation of CHK at Thr68. In addition, meriolin 16 induced the ATM-mediated phosphorylation of γH2AX (at Ser139) and KAP1 (at Ser824). However, in contrast to DNA damaging agents as the topoisomerase II inhibitor etoposide, inhibition of caspases completely blocked the meriolin 16 induced phosphorylation of γH2AX and KAP1. Thus, these data indicate that meriolin-inflicted DNA damage is rather an apoptotic downstream event mediated by caspases. This effect might be attributed to the caspase-mediated degradation of the inhibitor ICAD during apoptosis that subsequently releases and activates the caspase-activated DNase (CAD) which then produces the characteristic internucleosomal DNA cleavage [49, 50]. Apoptotic internucleosomal DNA cleavage might, in turn, activate ATM and induce the phosphorylation of γH2AX and KAP1, which in the case of meriolin 16, could be inhibited upon caspase-inhibition but not in case of direct DNA damage inflicted by etoposide. Thus, meriolin-induced activation of the mitochondrial apoptosis pathway is obviously not initiated upon DNA damage—as in the case of radio- and chemotherapy.

Bettayeb et al. could demonstrate that meriolin 3 inhibits the kinase activity of various cyclin-dependent kinases (such as CDK1–7 and CDK9) and prevents that CDK1, CDK4, or CDK9 phosphorylate their targets (e.g., retinoblastoma protein or RNA polymerase II). In addition, they observed that meriolin 3 induced the downregulation of the antiapoptotic Bcl-2 protein Mcl-1 [7]. Consequently, they proposed that meriolin 3-mediated inhibition of CDK9 reduces RNA polymerase II activity, which in turn leads to a reduction in transcriptional activity and thereby to the downregulation of short-lived protein transcripts, such as Mcl-1. These findings were further corroborated by Cidado et al., who showed that the selective CDK9-inhibitor AZD4573 induced a rapid downregulation of Mcl-1 mRNA, followed by a downregulation of Mcl-1 on protein level within 4 h, which preceded the onset of subsequent caspase-activation in the AML cell line MV-4-11 [51]. However, in contrast to Mcl-1, the protein expression of Bcl-2 and Bcl-xL remained rather stable within 9 h [51]. Similarly, meriolin 16, 31, or 36 did not affect the protein expression of Bcl-2 or Bcl-xL within 24 h (Suppl. Fig. 5). Thus, the data by Cidado et al. assign an exclusive role for Mcl-1 in apoptosis induction upon CDK9-inhibition [51]. This is further supported by the observation that overexpression of Mcl-1 reduces CDK-inhibitor-induced apoptosis [52, 53].

**Fig. 8:**
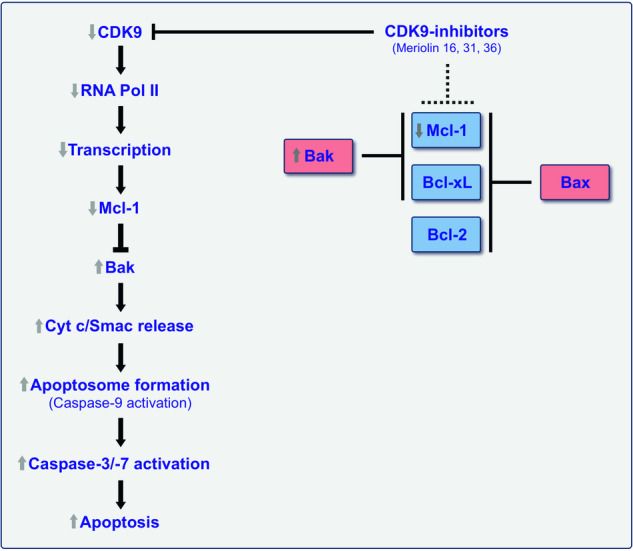
Schematic illustration of the potential mechanism of meriolin-induced apoptosis. Left panel: Besides CDKs involved in cell cycle regulation, meriolin 16 and 36 are also able to inhibit CDK9, which affects transcription rather than cell cycle control. CDK9 activates RNA polymerase II (RNA Pol II) via phosphorylation at Ser2. Inhibition of CDK9 has been shown to inhibit RNA polymerase II mediated transcription of the short-lived antiapoptotic Bcl-2 protein Mcl-1 [7, 51]. Downregulation of Mcl-1 then leads to the activation of proapoptotic proteins (such as Bak) that, in turn, mediate the mitochondrial release of proapoptotic cytochrome c and Smac. This event leads to the activation of initiator caspase-9 within the apoptosome and subsequent activation of effector caspase-3 and -7 and finally to apoptosis induction. Right panel: The antiapoptotic Bcl-2 proteins Bcl-2, Bcl-xL, and Mcl-1 display a different inhibitory potential towards the proapoptotic Bcl-2 proteins Bax and Bak. In contrast to Bcl-2, which only neutralizes Bax – Mcl-1 and Bcl-xL can inhibit Bax and Bak [54–56]. Since apoptosis induction mediated by CDK9-inhibitors seems to be primarily mediated by Bak (and not via Bax) [51], this might explain why meriolin-induced apoptosis is blocked in cells overexpressing Bcl-xL or Bax-/Bak-deficient cells, whereas Bcl-2 overexpressing cells are not affected. The scheme of the right panel was modified from [56].

However, the question still remains why overexpression of Bcl-2 has no effect on meriolin-induced apoptosis, while overexpression of Bcl-xL and Bax-/Bak-deficiency does. In this context, Cidado et al. could show that an siRNA double-knockdown of Bax and Bak completely abrogated CDK9-inhibitor-induced caspase-activation, whereas the knockdown of Bax reduced caspase-activation but not as potently compared to the Bak knockdown [51]. Thus Bak appears to be primarily responsible for apoptosis induction via CDK9-inhibition. In contrast to Bcl-2, which can only bind to Bax – Mcl-1 and Bcl-xL can interact with both Bax and Bak [54–56]. Since CDK-inhibition obviously induces apoptosis via Bak (due to the downregulation of short-lived Mcl-1), this might explain why overexpression of Bcl-2 does not affect meriolin-induced apoptosis (Fig. 8).

As we could show in a kinome screen that Meriolin 16 and 36 potently inhibited (with the exception of CDK4 and 6) almost all CDKs, including CDK9 (data not shown), the following scenario for meriolin-induced apoptosis is conceivable: in addition to CDKs involved in cell cycle regulation, meriolins also inhibit CDK9 that normally activates RNA polymerase II via phosphorylation at Ser2. This reduces the transcription of short-lived Mcl-1. Downregulation of Mcl-1 then shifts the balance towards activated Bak and subsequent mitochondrial release of cytochrome c and Smac, which upon apoptosome formation activate initiator caspase-9 and downstream located effector caspases 3 and 7 and ultimately apoptosis (Fig. 8).

Initially discovered in leukemia and lymphoma, antiapoptotic members of the Bcl-2 family, including Bcl-2, Bcl-xL, or Mcl-1, are frequently overexpressed in other neoplasia in order to block apoptosis in tumorigenesis. Furthermore, as the primary mechanism of genotoxic radio- and chemotherapy involves the p53-mediated activation of the mitochondrial death pathway, tumor cells develop therapy resistance by disabling this pathway e.g., via overexpression of antiapoptotic Bcl-2 proteins [33, 57]. Thus, the observation that meriolins are capable of activating the mitochondrial apoptosis pathway even in the presence of Bcl-2 renders them as valuable agents for the treatment of therapy-resistant Bcl-2-overexpressing tumors. This feature is corroborated by our observation that the tested meriolin derivatives were capable of inducing cell death in imatinib-resistant K562 and KCL22 CML cells as well as in cisplatin-resistant J82 urothelial carcinoma and 2102EP germ cell tumor cells (Fig. 7).

Besides cell death induction, it has also been shown that meriolins as multikinase inhibitors target kinases such as CDKs (e.g., CDK1–7 and CDK9) [7] and induce cell cycle arrest in G2/M-phase [11]. Thus, meriolins act as a double-edged sword, since, on the one hand, they inhibit CDKs (i.e., cell cycle progression and proliferation), and on the other hand, they can also induce cell death (i.e., apoptosis). This renders meriolins as valuable anticancer drugs since they simultaneously target two Achilles’ heels of the tumor—i.e., unlimited proliferation and inhibition of cell death [21, 22].

## Materials and methods

### Synthesis of meriolin 16, 31, and 36

The synthesis of meriolin 31 and meriolin 36 have been previously described [1]. Meriolin 16 has been synthesized in a similar fashion [8] and the experimental details and characterization are provided in the supplemental information.

### Reagents

Meriolin 3 (#445821) and carbonyl cyanide *m*-chlorophenyl hydrazone (CCCP, #C2759) were obtained from Sigma (Munich, Germany). Etoposide (#1043) was purchased from Biovision (Waltham, MA, USA), staurosporine (STS, #9300) from LC Laboratories (Woburn, MA, USA), and *N*-(2-Quinolyl)valyl-aspartyl-(2,6-difluorophenoxy)methyl ketone (Q-VD-OPh, QVD, #S7311) from Selleckchem (Houston, TX, USA). TRAIL (#ALX-201-115-C010) was purchased from Enzo Life Sciences (Farmingdale, NY, USA). All other substances for which a manufacturer is not explicitly specified were obtained from Carl Roth.

### Cell lines, primary cells, and cell culture

Jurkat cells (human T cell leukemia; #ACC-282) and DG75 cells (human B cell Burkitt lymphoma; #ACC-83) were obtained from DSMZ. Ramos cells (human Burkitt B cell lymphoma) were kindly provided by Michael Engelke (Institute of Cellular and Molecular Immunology, University Hospital Göttingen, Göttingen, Germany). HeLa cells (human cervix carcinoma) stably expressing mito-DsRed were kindly provided by Aviva M. Tolkovsky (Department of Clinical Neurosciences, University of Cambridge, England, UK) and have been previously described [58]. Caspase-9-deficient Jurkat cells were kindly provided by Klaus Schulze-Osthoff (Interfaculty Institute for Biochemistry, University of Tübingen, Germany) [59] and retrovirally transduced with either empty pMSCVpuro (Clontech, Heidelberg, Germany) or pMSCVpuro containing cDNAs coding for untagged human wild-type caspase-9 as previously described [37]. Jurkat cells expressing wild-type Bcl-2 and respective vector control cells were kindly provided by Claus Belka (Ludwig-Maximilians University, Munich, Germany) and previously described [60]. Caspase-8 deficient Jurkat cells and the parental cell line A3 were kindly provided by John Blenis (Sandra and Edward Meyer Cancer Center, New York, NY, USA) [36]. The Apaf-1-deficient human melanoma cell line SK-Mel-94 was kindly provided by Maria S. Soengas (Molecular Oncology Program, Spanish National Cancer Research Centre (CNIO), Madrid, Spain) [39].

Transient expression of Smac-mCherry was achieved by lipofection at 70–80% confluence using Lipofectamine 2000 (Life Technologies, Darmstadt, Germany). Briefly, HeLa cells were incubated with 0.15 μl Lipofectamine 2000, 50 ng pcDNA3-Smac(1–60)mCherry, and 25 μl optimem per well in glass bottom 8-well chambers (Ibidi, Planegg, Germany) for 16 h. The plasmid was kindly provided by Stephen Tait (Beatson Institute, University of Glasgow, Scotland, UK; plasmid Addgene ID 40880) and has been described previously [61]. The imatinib-sensitive and -resistant cell lines K562 (CML) and KCL22 (CML) were generated as previously described [42]. The cisplatin-sensitive and -resistant cell lines J82 (urothelial carcinoma) and 2102EP (germ cell tumor) were generated as previously described [44, 45]. HeLa, J82, Jurkat, K562, KCL22, Ramos, and SK-Mel-94 cells were cultivated in DMEM or RPMI medium supplemented with 10% FCS, 100 U/ml penicillin, 100 µg/ml streptomycin, and 10 mM HEPES at 37 °C and 5% CO_2_ in a humidity-saturated atmosphere. 2102EP germ cell tumor cells were cultivated in DMEM (1x) plus GlutaMAX-I medium supplemented with 10% FBS, 1% penicillin/streptomycin (10,000 U), and 1% l-glutamin (200 mM) at 37 °C and 7.5% CO_2_ and kindly provided by Christoph Oing (University Medical Center Hamburg-Eppendorf, Germany) and Friedemann Honecker (Tumor and Breast Center ZeTuP, St. Gallen, Switzerland) [43].

### CRISPR/Cas-based knockdown of Apaf-1 in Jurkat cells

Stable transcription repression of Apaf-1 was achieved using the CRISPR inhibition system. The plasmid pLVhU6-sgRNA hUbc-dCas9-KRAB-T2a-Puro (Addgene #71236, a gift from Charles Gersbach) [62] was utilized. The guide RNAs (sgRNA) were designed using the CRISPR Design service engine (http://crispr.mit.edu). The sgRNA (GGACGTGACTGCTCTATCCC) targeting the exon 1 of Apaf-1 was used to achieve stable knockdown (Apaf-1-KD), while plasmid carrying non-targeted sequence (GTTCCGCGTTACATAACTTA) was used to generate control cells (Apaf-1-Ctrl). Lentivirus was produced by transfecting HEK293T with packaging vectors (pMDLg/pRRE (Addgene #12251), pRSV-Rev (Addgene #12253), and pMD2.G (Addgene #12259), gifts from Didier Trono [63]). Pure populations of the stable cell lines were selected using 2 µg/ml puromycin (InvivoGen, Toulouse, France). The stable knockdown of Apaf-1 was examined using quantitative reverse transcription PCR (qRT-PCR). After RNA was extracted from manipulated cells using the SV total RNA isolation system (Promega, Mannheim, Germany), extracted RNA (1–3 μg) was reverse transcribed with M-MLV reverse transcriptase (Promega). The resulting cDNA was then amplified by qRT-PCR. To reduce the risk of false positives caused by the amplification of any contaminating genomic DNA, PCR primers were designed to span exon-exon junction (forward: TCTTCCAGTGGTAAAGATTCAGTT, reverse: AAACAACTGGCCTCTGTGGT).

### Cytotoxicity measurements

For the measurement of cell viability in Ramos cells, the resazurin reduction assay (also known as AlamarBlue® assay) was performed. In short, cells were seeded at a certain density depending on the intended incubation time (5 × 10^5^ cells/well for 8 or 24 h, 1 × 10^5^ cells/well for 72 h), treated with ascending substance concentrations, and after the specified incubation time, resazurin (Sigma, #R7017) was added to a final concentration of 40 µM. After 120 min of incubation, the fluorescence of resorufin (excitation: 560 nm, emission: 590 nm) was measured with a microplate spectrophotometer. Since the reduction of resazurin to resorufin is proportional to aerobic respiration, it serves as a measure of cell viability. Alternatively, the triphenyl tetrazolium chloride (XTT) assay was used to evaluate the cell viability of 2102EP germ cell tumor cells as previously described [64].

### Fluorometric caspase-3 activity assay

Ramos or Jurkat cells were seeded at a density of 1 × 10^6^ cells/ml and treated with the respective agents for the indicated time. Briefly, cells were harvested by centrifugation at 600×*g* and lysed with 50 μl of ice-cold lysis buffer (20 mM HEPES, 84 mM KCl, 10 mM, MgCl_2_, 200 μM EDTA, 200 μM EGTA, 0.5% NP40, 1 μg/ml leupeptin, 1 μg/ml pepstatin, 5 μg/ml aprotinin) on ice for 10 min. Cell lysates were transferred to a flat-bottom microplate and mixed with 150 μl of ice-cold reaction buffer (50 mM HEPES, 100 mM NaCl, 10% sucrose, 0.1% CHAPS, 2 mM CaCl_2_, 13.35 mM DTT) containing 50 μM of the profluorescent caspase substrate Ac-DEVD-AMC (Biomol GmbH, Hamburg, Germany, #ABD-13402). The kinetics of AMC release were monitored by measuring AMC fluorescence intensity (excitation: 360 nm, emission: 450 nm) at 37 °C in intervals of 2 min over a time course of 120 min, using a Synergy Mx microplate reader. The slope of the linear range of the fluorescence curves (Δrfu/min) was considered as corresponding to caspase-3 activity. Therefore, the mean slope of DMSO-treated wells per time point and cell line was set to 100 and all other values were normalized to the corresponding DMSO mean.

### FACS-based analysis of apoptotic cell death

The leakage of fragmented DNA from apoptotic nuclei was measured by the method of Nicoletti et al. [25]. Briefly, nuclei were prepared by lysing cells in a hypotonic lysis buffer (1% sodium citrate, 0.1% Triton X-100, 50 µg/ml propidium iodide) and subsequently analyzed by flow cytometry. Nuclei to the left of the 2 N peak containing hypodiploid DNA were considered as apoptotic. All flow-cytometric analyses were performed on an LSR-Fortessa™ (Becton Dickinson, Heidelberg, Germany).

### Immunoblotting

Ramos or Jurkat cells were treated as specified and then harvested by centrifugation (900×*g*, 5 min) and quick-frozen in liquid nitrogen. Cell pellets were thawed on ice, incubated in lysis buffer [20 mM Tris-HCl, 150 mM NaCl, 1% v/v Triton X-100, 0.5 mM EDTA, 1 mM Na_3_VO_4_, 10 mM NaF, 2.5 mM Na_4_P_2_O_7_, 0.5% sodium deoxycholate, protease inhibitors (Sigma, #P2714)] for 30 min. Cell lysates were purified from cell debris by centrifugation (17,000×*g*, 15 min, 4 °C) and the protein concentration in the supernatant was determined by Bradford assay. The samples were diluted to a homogeneous protein concentration with sample buffer and both SDS-PAGE and Western blot analyses were performed. Finally, target protein specific primary antibodies [anti-Bak (Becton Dickinson, #556382), anti-Bax (Becton Dickinson, #610982), anti-Bcl-2 (Santa Cruz Biotechnology, #509), anti-Bcl-xL (Cell Signaling S4H6, #27645), anti-Caspase-8 (Cell Signaling Technology, #9746), anti-caspase-9 (Thermo Fisher, #PA5-16358), anti-GAPDH (glyceraldehyde 3-phosphate dehydrogenase; Abcam, #ab8245), anti-OPA1 [65], anti-PARP1 (Enzo, #BML-SA250), anti-tubulin (TUBA4A; Sigma, #T5168), or anti-vinculin (Sigma, #V4139)], were applied and fluorescence-coupled secondary antibodies (LI-COR Biosciences) were used for protein detection on a PVDF membrane using the LI-COR Odyssey® imaging system.

### Microscopy

Imaging of HeLa cells stably expressing mito-DsRed was performed using an Axio Observer 7 inverted microscope (Zeiss) equipped with a Plan-Apochromat 40x/1.4 Oil DIC (UV) VIS-IR M27 oil-immersion objective and a Colibri7 LED light source and using optical sectioning via Zeiss Apotome.2. The cells were seeded on glass bottom eight-well chamber slides (Ibidi, Planegg, Germany) and maintained in full growth medium supplemented with 10 mM HEPES during imaging. Images were taken once per hour, and between measurements, cells were incubated at 37 °C.

Confocal microscopy live imaging of Hela cells transiently expressing Smac-mCherry was performed using a SP8 Leica microscope (Inverse DMI 6000 AFC, Leica) with a C‐Apochromat × 63 N.A. 1.2 water DIC objective. Cells were seeded onto µ-Slide eight wells (Ibidi, #80826) and transfected with Smac-mCherry as described before [66]. Cells were maintained in full growth medium at 37 °C and 5% CO_2_ during imaging and recorded every 30 min for 300 min with z-stack acquisition for correct signal detection. Images were analyzed and processed, including smoothing and ROI detection at individual frames with Fiji [67]. For each individual time frame, the standard deviation (SD) of the fluorescence intensity of Smac-mCherry was measured to monitor its subcellular distribution upon meriolin treatment (1 μM) supplemented with 10 μM QVD. A low SD is associated with a homogenous distribution (cytosolic), whereas a high SD is related to a confined accumulation in the mitochondria.

### Measurement of mitochondrial membrane potential

To measure changes in mitochondrial membrane potential (ΔΨm), Ramos or Jurkat cells were loaded with the cell-permeable and positively charged dye tetramethylrhodamine ethyl ester (TMRE), which accumulates in active mitochondria characterized by a negative net charge, whereas depolarized mitochondria do not retain the dye. For this purpose, cells were resuspended in fresh medium containing 10 mM HEPES and supplemented with 100 nM TMRE (AAT Bioquest, Sunnyvale, CA, USA; #22220). After incubation for 15 min at 37 °C, cells were washed twice with RPMI medium (plus HEPES) and incubated for another 15 min in order to allow the cells to recover and to ensure that the dye had accumulated in active mitochondria. Subsequently, the fluorescence of TMRE was measured by flow cytometry (excitation: 488 nm, emission: 575) with an LSR-Fortessa™ (Becton Dickinson, Heidelberg, Germany). Treatment with the protonophore CCCP served as a positive control for complete mitochondrial depolarization.

### Replicates and statistical analyses

Experiments were replicated at least three times, and representative data are shown, unless otherwise stated. Error bars indicate standard deviation. All statistical analyses were performed using Prism v7.01 (GraphPad Software, La Jolla, CA, USA).

### Supplementary information

Supplemental Figures, Supplemental Materials and Methods, Supplemental References

Original Data File

## Data Availability

Data were generated by the authors and included in the article.
