# Supplementary material for: Novel meriolin derivatives activate the mitochondrial apoptosis pathway in the presence of antiapoptotic Bcl-2
Source: Cell Death Discov. 2024 Mar 9;10:125. doi: 10.1038/s41420-024-01901-y (PMC10924942; doi:10.1038/s41420-024-01901-y)
Supplement: Supplementary file 1 — Supplemental Figures, Supplemental Materials and Methods, Supplemental References [file 41420_2024_1901_MOESM1_ESM.pdf]

Supplemental Information

Supplemental Figures

Supplemental Figure 1: Cytotoxicity of meriolin 3 in Ramos lymphoma cells

Supplemental Figure 2: Meriolin-induced DNA damage and checkpoint activation is a downstream event mediated by caspases

Supplemental Figure 3: Meriolin-induced phosphorylation of CHK2 at Thr68 is abolished in the presence of the pan-caspase inhibitor QVD

Supplemental Figure 4: Meriolin 16, 31 and 36 induced apoptosis is not affected by overexpression of Bcl-2 but is blocked in presence of Bcl-xL and in the absence of Bax/Bak or Apaf-1

Supplemental Figure 5: Protein expression of Bcl-2 and Bcl-xL in Jurkat cells is not affected upon meriolin 16, 31 and 36 induced apoptosis

Supplemental Figure 6: ^{1}H NMR spectrum of 3-iodo-4-methoxy-1-tosyl-1H-pyrrolo[2,3-b]pyridine (DMSO-d_6, 600 MHz, T = 298 K)

Supplemental Figure 7: ^{13}C NMR spectrum of 3-iodo-4-methoxy-1-tosyl-1H-pyrrolo[2,3-b]pyridine (DMSO-d_6, 150 MHz, T = 298 K)

Supplemental Figure 8: ^{1}H NMR spectrum of meriolin 16 (DMSO-d_6, 600 MHz, T = 298 K)

Supplemental Figure 9: ^{13}C NMR spectrum of meriolin 16 (DMSO-d_6, 150 MHz, T = 298 K)

Supplemental Materials and Methods

Synthesis of 3-Iodo-4-methoxy-1-tosyl-1*H*-pyrrolo[2,3-b]pyridine

Supplemental References

Supplemental Figures

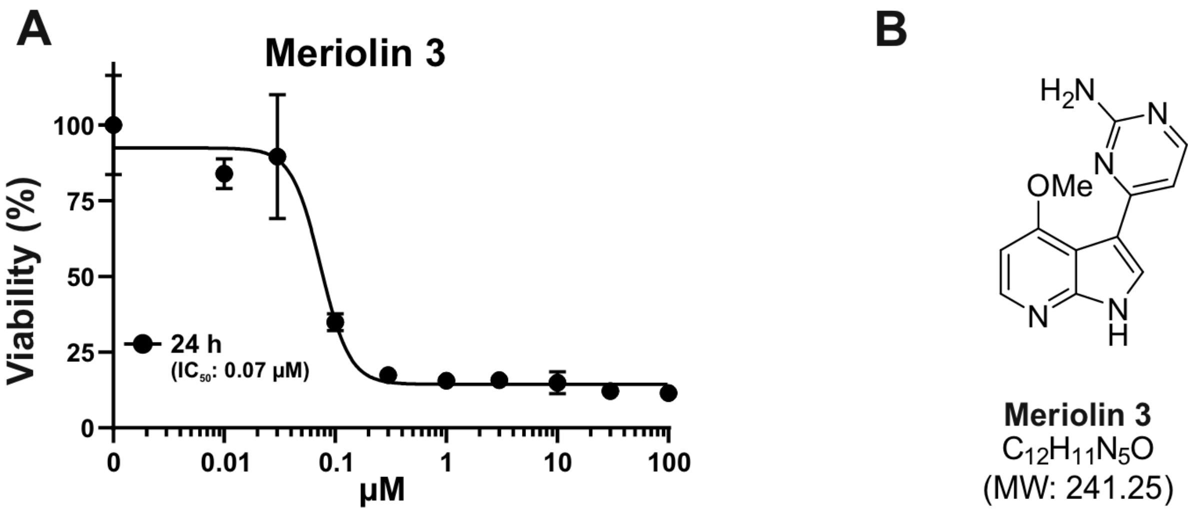

Suppl. Figure 1: Cytotoxicity of meriolin 3 in Ramos lymphoma cells.

(A) 5 x 10^5 Ramos Burkitt B cell lymphoma cells were treated with increasing concentrations of meriolin 3. After 24 h, viability was determined by AlamarBlue® assay. The respective IC_{50} value is given in parenthesis. Error bars = SD of triplicates. **(B)** Structure of meriolin 3.

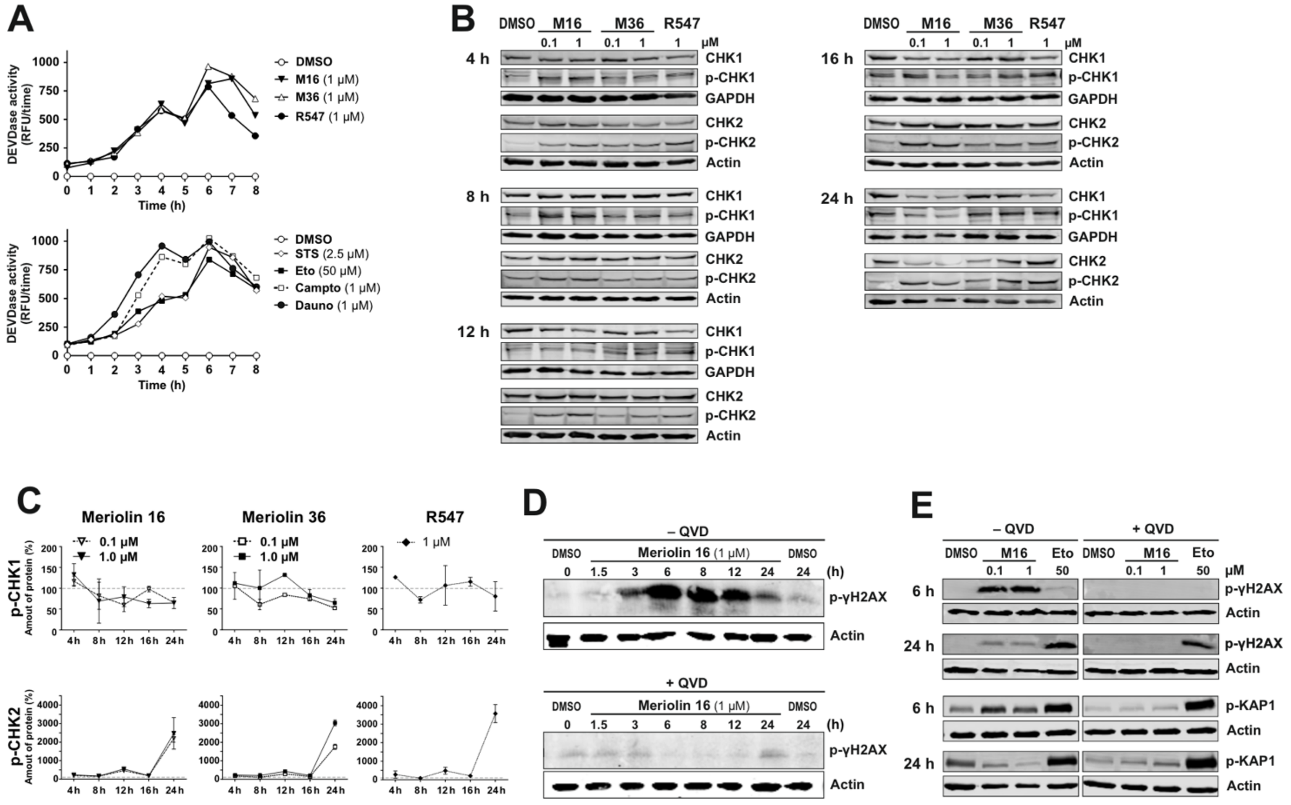

Suppl. Figure 2: Meriolin-induced DNA damage and checkpoint activation is a downstream event mediated by caspases.

(A) Ramos Burkitt B cell lymphoma cells were treated with 0.1% v/v DMSO (diluent control), 1 µM meriolin 16 (M16), 1 µM meriolin 36 (M36), 1 µM R547, 2.5 µM staurosporine (STS), 50 µM etoposide (Eto), 1 µM camptothecin (Campto), or 1 µM daunorubicin (Dauno) in a kinetics up to 8 h. The topoisomerase inhibitors etoposide, camptothecin and daunorubicin were applied as DNA-damaging agents. The activation of caspase-3 was determined by fluorometric analysis of DEVDase activity. Shown in each graph is the mean ± SD of one representative experiment performed in duplicates. **(B)** Ramos cells were treated with DMSO (0.1% v/v), 0.1 µM or 1 µM of meriolin 16 or 36, and the CDK-inhibitor R547 for up to 24 h. The proteins of interest were analyzed via immunoblotting: CHK1, phospho-Ser345-CHK1 (p-CHK1), CHK2 and phospho-Thr68-CHK2 (p-CHK2). GAPDH (glyceraldehyde 3-phosphate dehydrogenase) and β-actin served as loading controls. For each time point the respective protein level was analyzed via immunoblotting and subsequently quantified. Shown is one set of representative immunoblots from 4 h, 8 h, 12 h, 16 h and 24 h. **(C)** The respective quantification of the protein levels (n ≥ 2) of p-Ser345-CHK1 (p-CHK1) and p-Thr68-CHK2 (p-CHK2) upon meriolin 16, meriolin 36 or R547 treatment are shown as a graph over time. **(D)** Ramos cells were incubated in the presence or absence of the pan-caspase inhibitor QVD (10 µM) and subsequently further treated with DMSO (0.1% v/v) or 1 µM Meriolin 16 for 24 h. The protein level of phospho-Ser139-γH2AX (p-γH2AX) was analyzed via immunoblotting and β-actin served as loading control. **(E)** Ramos cells were pretreated with or without QVD (10 µM) and subsequently further incubated with DMSO (0.1% v/v), 0.1 or 1 µM meriolin 16 (M16) or 50 µM etoposide (Eto; positive control for DNA damage induction) for 6 or 24 h. **Upper immunoblot:** The protein level of p-Ser139-γH2AX was analyzed via immunoblotting and β-actin served as loading control. **Lower immunoblot:** The protein level of phospho-Ser824-KAP1 (p-KAP1) was analyzed via immunoblotting and β-actin used as loading control.

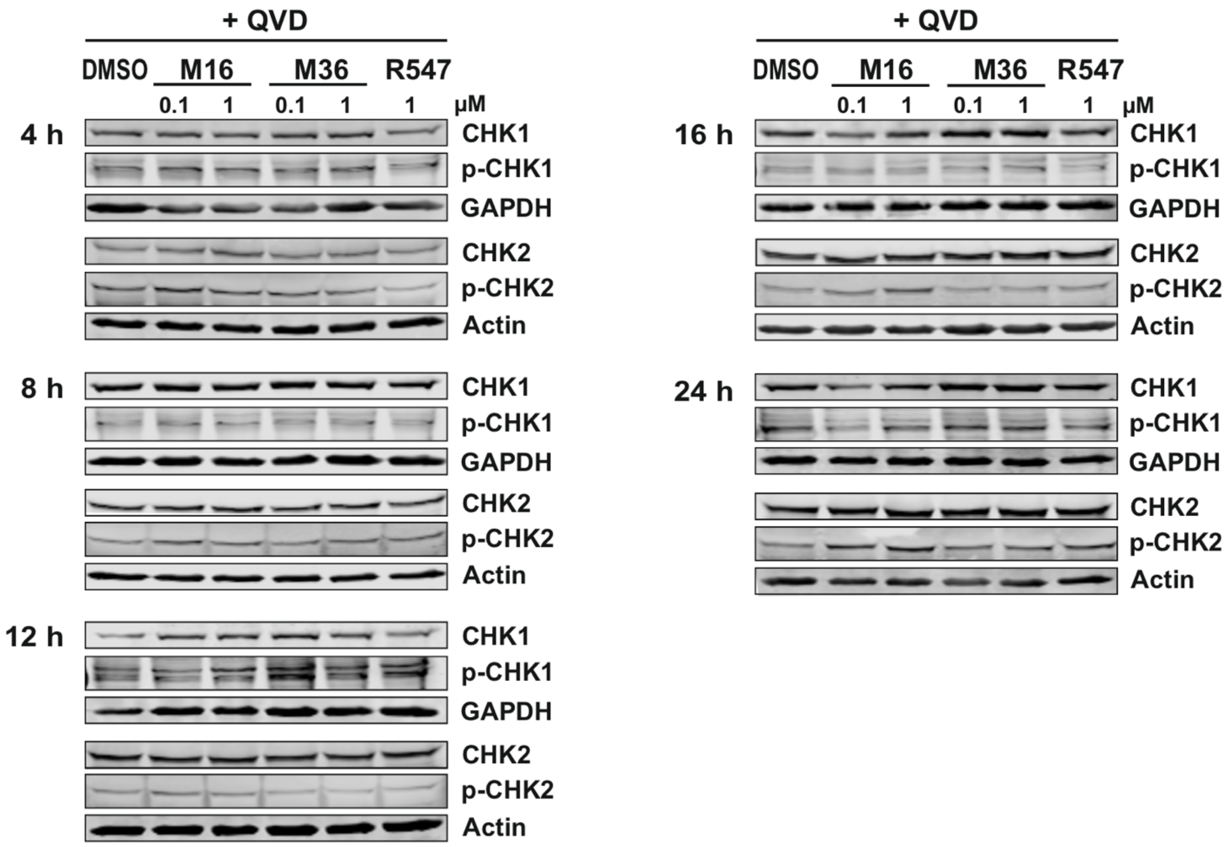

Suppl. Figure 3: Meriolin-induced phosphorylation of CHK2 at Thr68 is abolished in the presence of the pan-caspase inhibitor QVD.

Ramos cells were incubated in the presence or absence of the pan-caspase inhibitor QVD (10 µM) and subsequently further treated (as in Suppl. Figure 2) with DMSO (0.1% v/v), 0.1 µM or 1 µM of meriolin 16 or 36, and the CDK-inhibitor R547 for up to 24 h. The proteins of interest were analyzed via immunoblotting: CHK1, phospho-Ser345-CHK1 (p-CHK1), CHK2 and phospho-Thr68-CHK2 (p-CHK2). GAPDH and β-actin served as loading controls. For each time point the respective protein level was analyzed via immunoblotting. Shown is one set of representative immunoblots from 4 h, 8 h, 12 h, 16 h and 24 h.

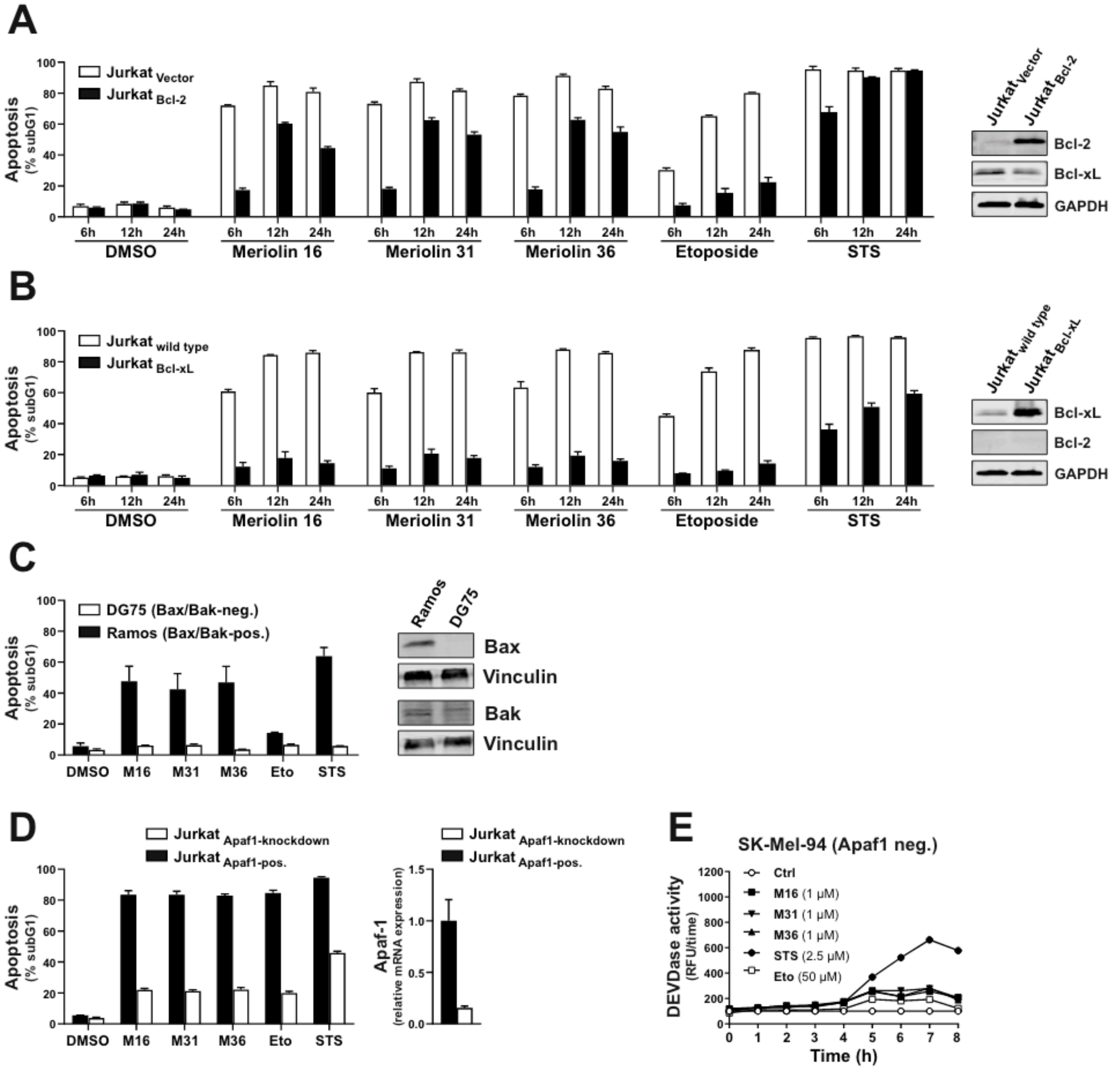

Suppl. Figure 4: Meriolin 16, 31 and 36 induced apoptosis is not affected by overexpression of Bcl-2 but is blocked in presence of Bcl-xL and in the absence of Bax/Bak or Apaf-1.

(A) Meriolin 16, 31 and 36 induce apoptosis in the presence of antiapoptotic Bcl-2. Jurkat cells stably transfected with vectors encoding Bcl-2 (Jurkat-Bcl-2; black bars) [1] or empty vector (Jurkat-vector; white bars) were treated as in Figure 4C, D with 0.1% (v/v) DMSO, 1 µM of meriolin 16, 31 and 36, 50 µM etoposide (Eto), or 2.5 µM staurosporine (STS) for 6 h, 12 h or 24 h. After indicated time points, apoptosis was assessed by flow cytometric measurement of apoptotic hypodiploid nuclei. **(A, B)** Immunoblots of expression level of Bcl-2 and Bcl-xL are shown in right panel. **(B)** Meriolin 16, 31 and 36 induced apoptosis is blocked in presence of antiapoptotic Bcl-xL. Jurkat cells stably transfected with vectors encoding Bcl-xL (Jurkat-Bcl-xL; black bars) [2] or wild type cells (Jurkat-wild type; white bars) were treated as in Suppl. Figure 4A). After indicated time points, apoptosis was assessed by flow cytometric measurement of apoptotic hypodiploid nuclei. **(C)** Meriolin 16, 31 and 36 induced apoptosis is blocked in the Bax- and Bak-deficient human B cell Burkitt lymphoma cell line DG75 [3, 4]. Bax/Bak-deficient DG75 cells and Bax/Bak-expressing Ramos cells (human B cell Burkitt lymphoma cells; used as positive control) were treated with 0.1% (v/v) DMSO (solvent control), 1 µM of meriolin 16, 31 and 36, 50 µM of etoposide (Eto), 2.5 µM staurosporine (STS). After

24 h, apoptosis was assessed by propidium iodide staining of apoptotic hypodiploid nuclei and flow cytometry. Expression level of Bax and Bak is shown in right panel. **(D, E)** Meriolin-induced apoptosis requires Apaf-1. **(D)** The CRISPR inhibition system was used to achieve a stable knockdown of Apaf-1. Apaf-1 proficient (Jurkat-Apaf1-pos.; black bars) and Apaf-1 knockdown Jurkat cells (Jurkat-Apaf1-knockdown; white bars) were treated as in Suppl. Figure 4C. After 24 h, apoptosis was assessed by flow cytometric measurement of apoptotic hypodiploid nuclei. The relative mRNA expression level of Apaf-1 is shown in the right panel, with error bars indicating the standard error of the mean (SEM). **(E)** Apaf-1 deficient SK-Mel-94 melanoma cells [5] were treated with 0.1% (v/v) DMSO, 1 µM of meriolin 16, 31 and 36, 50 µM etoposide (Eto), or 2.5 µM staurosporine (STS). Caspase-3 activity was measured after the indicated time period via measurement of DEVD-AMC fluorescence. **(A-D)** Immunoblotting for GAPDH (A, B, D) or vinculin (C) served as loading control.

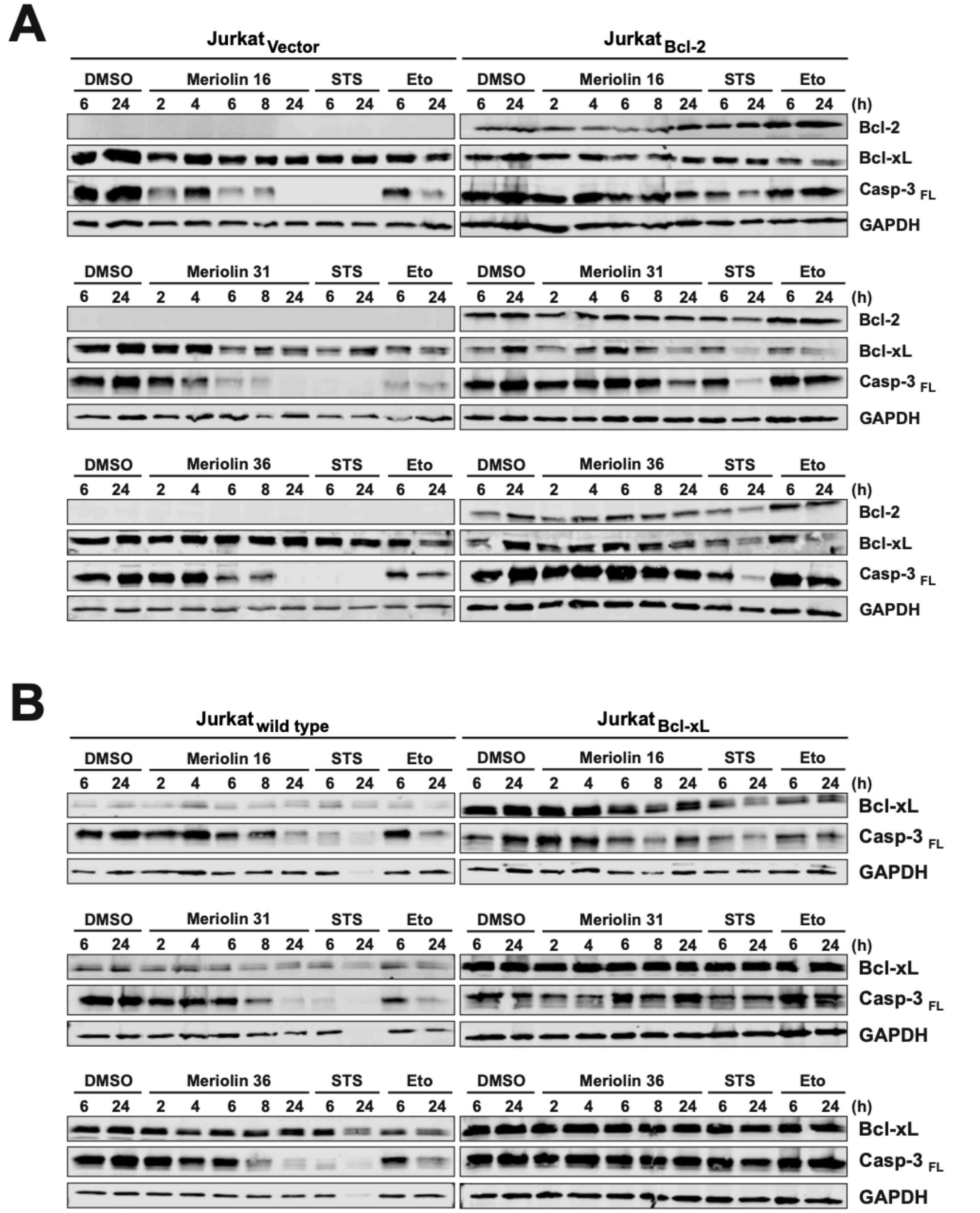

Suppl. Figure 5: Protein expression of Bcl-2 and Bcl-xL in Jurkat cells is not affected upon meriolin 16, 31 and 36 induced apoptosis.

(A) Jurkat cells stably transfected with vectors encoding Bcl-2 (Jurkat-Bcl-2) [1] or empty vector (Jurkat-vector) or **(B)** Jurkat cells stably transfected with vectors encoding Bcl-xL (Jurkat-Bcl-xL) [2] or wild type cells (Jurkat-wild type) were treated as in Suppl. Figure 4A, B with 0.1% (v/v) DMSO, 50 µM etoposide (Eto), or 2.5 µM staurosporine (STS) for 6 h or 24 h or with 1 µM of meriolin 16, 31 and 36 for 2 h, 4 h, 6 h, 8 h or 24 h. After indicated time points, immunoblots against Bcl-2 (A), or Bcl-xL (A, B) were performed. Immunoblotting against Bcl-2 in Jurkat Bcl-xL and Jurkat wild type cells (B) was omitted since these cells were Bcl-2 deficient (as shown in Suppl. Figure 4B). Activation of caspase-3 represented by the loss of the 32 kDa full length form of caspase-3 (Casp-3 FL) was also monitored. Immunoblotting for GAPDH was used as loading control.

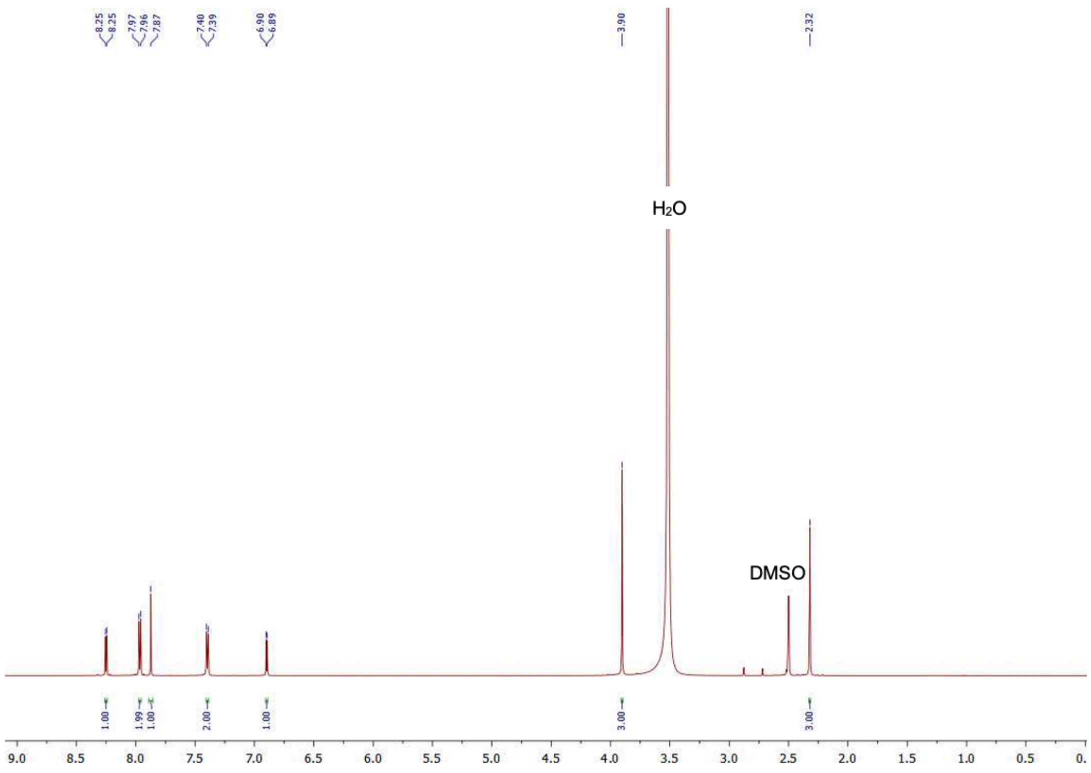

Suppl. Figure 6: ^{1}H NMR spectrum of 3-iodo-4-methoxy-1-tosyl-1H-pyrrolo[2,3-b]pyridine (DMSO-d$_6$, 600 MHz, *T* = 298 K).

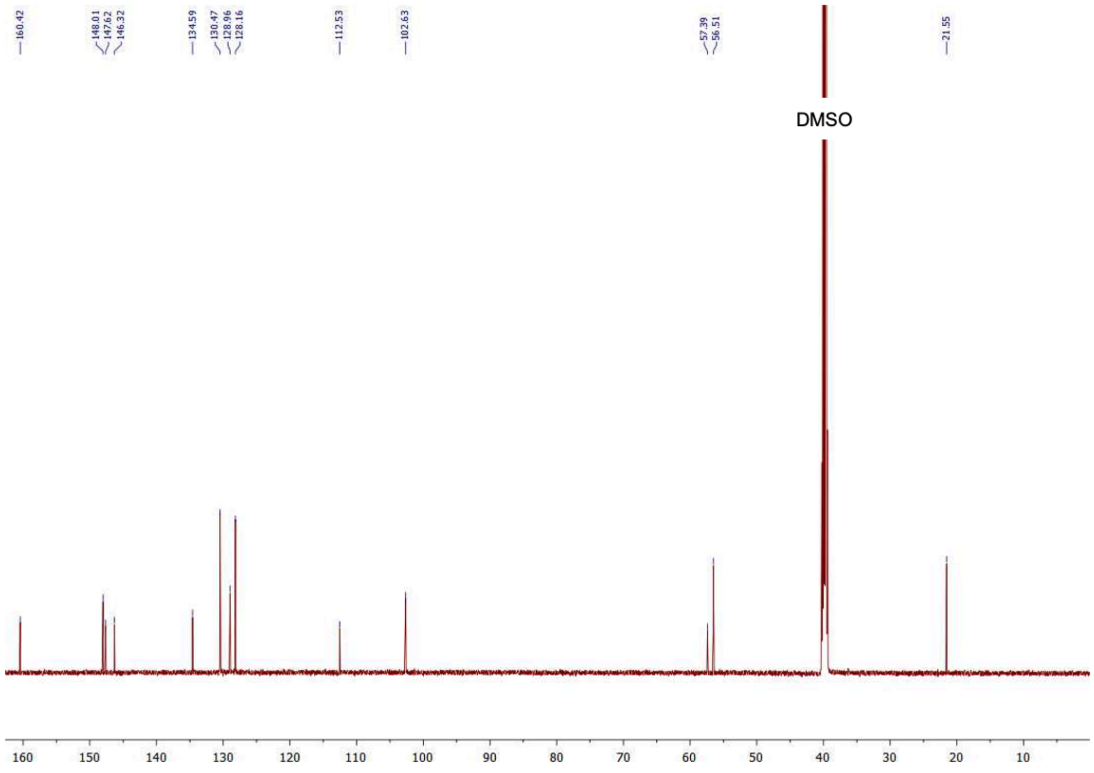

Suppl. Figure 7: ^{13}C NMR spectrum of 3-iodo-4-methoxy-1-tosyl-1H-pyrrolo[2,3-b]pyridine (DMSO-d$_6$, 150 MHz, *T* = 298 K).

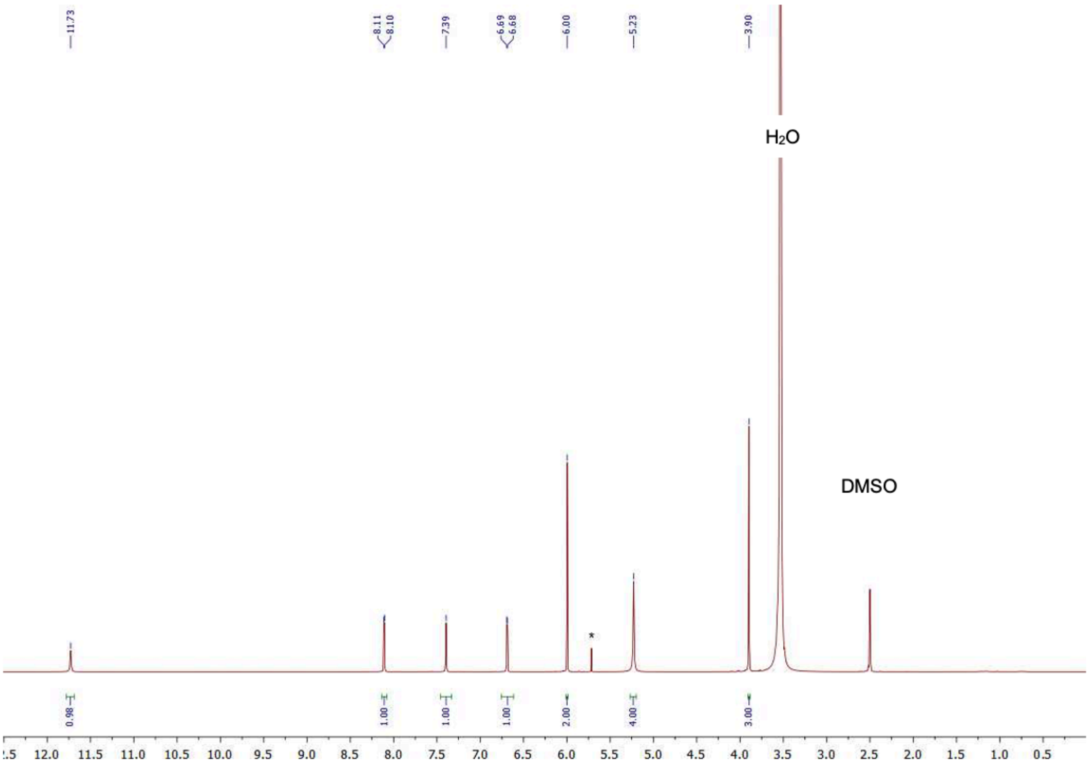

Suppl. Figure 8: ^{1}H NMR spectrum of meriolin 16 (DMSO-d$_6$, 600 MHz, *T* = 298 K), *residual solvent.

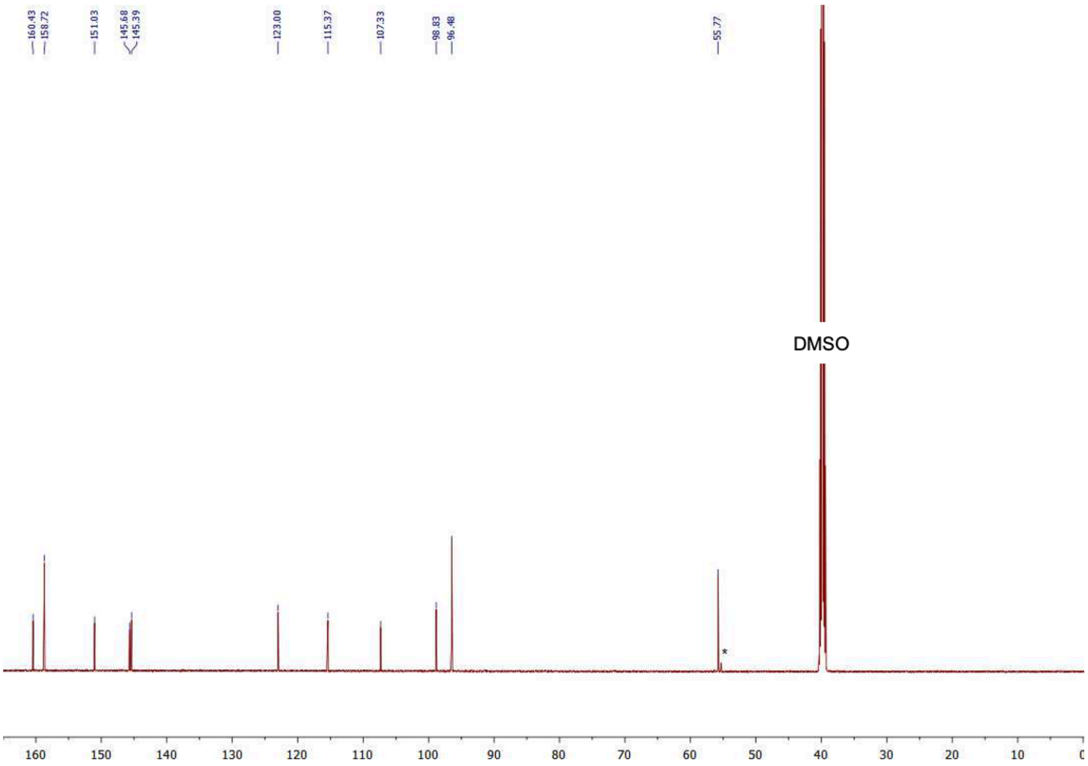

Suppl. Figure 9: ^{13}C NMR spectrum of meriolin 16 (DMSO-d$_6$, 150 MHz, *T* = 298 K), *residual solvent.

Supplemental Materials and Methods

Synthesis of 3-Iodo-4-methoxy-1-tosyl-1*H*-pyrrolo[2,3-*b*]pyridine [6]

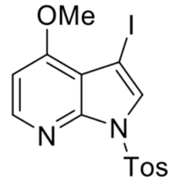

2g
$C_{15}H_{13}IN_2O_3S$
(MW: 428.24)

In an oven-dried 250 ml two-necked round bottom flask with magnetic stir bar were placed 4-methoxy-7-azaindole (0.74 g, 5.00 mmol), potassium hydroxide (0.70 g, 12.5 mmol) and dry DMF (30.0 ml). To this solution was added dropwise a solution of iodine (1.28 g, 5.05 mmol) in dry DMF (30.0 ml) at room temperature over 10 min. Then, the reaction mixture was stirred at room temperature for 30 min. After addition of another portion of potassium hydroxide (0.70 g, 12.5 mmol) and stirring at room temperature for 10 min, a solution of *p*-toluenesulfonylchloride (2.00 g, 10.5 mmol) in dry DMF (30.0 ml) was added dropwise over 20 min. After stirring at room temperature for 2.5 h the reaction mixture was cooled with an external ice-water bath and ice water (150 ml) was added dropwise from a dropping funnel. The precipitate was filtered to give after drying under vacuo to weight constancy 3-iodo-4-methoxy-1-tosyl-1H-pyrrolo[2,3-b]pyridine (2.02 g (4.70 mmol, 94 %) as a light yellow solid, Mp 164.4-165.4 °C, R_f(H:EE = 3:1) = 0.34. The ^{1}H NMR spectrum of 3-iodo-4-methoxy-1-tosyl-1*H*-pyrrolo[2,3-*b*]pyridine is depicted in Suppl. Figure 6, and the ^{13}C NMR spectrum of 3-iodo-4-methoxy-1-tosyl-1*H*-pyrrolo[2,3-*b*]pyridine is depicted in Suppl. Figure 7.

^{1}H NMR (DMSO-d$_6$, 300 MHz): δ = 2.32 (s, 3 H, C*H*$_3$), 3.90 (s, 3 H, OC*H*$_3$), 6.90 (d, $^3J_{HH}$ = 5.7 Hz, 1 H, C*H*$_{Ar}$), 7.40 (d, $^3J_{HH}$ = 8.4 Hz, 2 H, C*H*$_{Ar}$), 7.87 (s, 1 H, C*H*$_{Ar}$), 7.97 (d, $^3J_{HH}$ = 8.4 Hz, 2 H, C*H*$_{Ar}$), 8.25 (d, $^3J_{HH}$ = 5.7 Hz, 1 H, C*H*$_{Ar}$). ^{13}C NMR (DMSO-d$_6$, 75 MHz): δ = 21.5 (*C*H$_3$), 56.5 (O*C*H$_3$), 57.4 (*C*$_{quat}$), 102.6 (*C*H), 112.5 (*C*$_{quat}$), 128.2 (CH), 129.0 (*C*H), 130.5 (*C*H), 134.6 (*C*$_{quat}$), 146.3 (*C*$_{quat}$), 147.6 (*C*$_{quat}$), 148.0 (*C*H), 160.4 (*C*$_{quat}$). EI MS (*m/z* (%)): 428 (46) [M$^+$], 365 (15), 364 (100) [$C_{15}H_{13}IN_2O^+$], 274 (15), 273 (100) [$C_8H_6IN_2O^+$], 243 (22), 237 (12), 155 (13) [$C_7H_7O_2S^+$], 131 (23), 116 (62) [$C_7H_4N_2^+$], 91 (73) [$C_7H_7^+$], 65 (22) [$C_5H_5^+$]. IR (neat): $\tilde{\nu}$ [cm^{-1}]: 627 (s), 664 (s), 685 (s), 702 (m), 721 (s), 764 (w), 787 (m), 799 (w), 808 (s), 868 (w), 955 (w), 1015 (m), 1043 (w), 1088 (m), 1119 (s), 1163 (m), 1180 (s), 1215 (m), 1248 (w), 1269 (w), 1290 (m),1312 (w), 1325 (m), 1360 (w), 1371 (s), 1398 (w), 1435 (w), 1452 (w), 1460 (w), 1489 (m), 1551 (w), 1572 (m), 1595 (m), 1676 (w), 1894 (w), 2778 (w), 2845 (w), 2887 (w), 2903 (w), 2949 (w), 2986 (w), 3034 (w), 3065 (w), 3096 (w), 3142 (w).

Synthesis of 4-(4-methoxy-1*H*-pyrrolo[2,3-*b*]pyridin-3-yl)pyridin-2,6-diamine (meriolin 16)

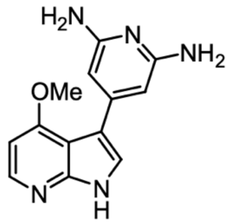

Meriolin 16
$C_{13}H_{13}N_5O$
(MW: 255.28)

In an oven-dried Schlenk flask with magnetic stir bar were placed 3-iodo-4-methoxy-1-tosyl-1H-pyrrolo[2,3-b]pyridine, (428 mg, 1.00 mmol) tetrakis(triphenylphosphane)palladium(0) (35.0 mg, 0.03 mmol) and dry 1,4-dioxane (5.00 ml) under argon. The solution was purged with constant stream of argon for 10 min. Then, dry triethylamine (1.40 ml, 10.0 mmol) and pinacolborane (0.25 ml, 1.70 mmol) were added to the reaction mixture. The reaction mixture was heated in the sealed Schlenk flask at 80 °C (preheated oil bath) for 4 h. After cooling of the reaction mixture to room temperature in an external water bath, methanol (5.00 ml) was added to the reaction mixture under argon atmosphere. After stirring at room temperature for 10 min, 4-bromopyridine-2,6-diamine (188 mg, 1.00 mmol; purchased from Sigma-Aldrich and used without further purification) and cesium carbonate (823 mg, 2.50 mmol) were successively added to the reaction mixture under argon. The reaction mixture was heated in the sealed Schlenk flask at 100 °C (preheated oil bath) for 20 h. After cooling to room temperature micro granules of NaOH (100 mg, 2.50 mmol) were added to the reaction mixture. The reaction mixture was heated in the sealed Schlenk flask at 100 °C (preheated oil bath) for 4 h. After cooling to room temperature the reaction mixture was absorbed on celite® and the solvent were removed in vacuo, followed by flash chromatography on silica gel (dichloromethane/methanol:/aqueous ammonia 100:8:1). After evaporation of the solvents in vacuo the crude product was triturated with pentane, the supernatant was discarded and the residue was dried under high vacuum at 70 °C for 48 h to give meriolin 16 (250 mg, 98%) as a colorless solid, Mp 255.0-259.8 °C (dec.), R_f(CH_2Cl_2/MeOH/NH_3 = 100:10:1) = 0.30. The ^{1}H NMR spectrum of meriolin 16 is depicted in Suppl. Figure 8, and the ^{13}C NMR spectrum of meriolin 16 is depicted in Suppl. Figure 9.

^{1}H NMR (DMSO-d$_6$, 600 MHz): δ = 3.90 (s, 3 H, OCH_3), 5.23 (s, 4 H, NH_2), 6.00 (s, 2 H, CH_{Ar}), 6.69 (d, $^3J_{HH}$ = 5.5 Hz, 1 H, CH_{Ar}), 7.39 (s, 1 H, CH_{Ar}), 8.11 (d, $^3J_{HH}$ = 5.5 Hz, 1 H, CH_{Ar}), 11.73 (s, 1 H, NH). ^{13}C NMR (DMSO-d$_6$, 150 MHz): δ = 55.8 (OCH_3), 96.5 (*C*H), 98.8 (*C*H), 107.3

(C_{quat}), 115.4 (C_{quat}), 123.0 (*C*H), 145.4 (*C*H), 145.7 (C_{quat}), 151.0 (C_{quat}), 158.7 (C_{quat}), 160.4 (C_{quat}). IR (neat): $\tilde{\nu}$ [cm^{-1}]: 617 (m), 640 (s), 664 (m), 685 (m), 712 (m), 739 (m), 779 (w), 801 (s), 833 (m), 847 (m), 941 (w), 970 (w), 1028 (w), 1057 (w), 1088 (m), 1146 (m), 1181 (w), 1200 (w), 1234 (w), 1248 (m), 1279 (m), 1290 (w), 1317 (m), 1339 (w), 1362 (w), 1393 (m), 1429 (m), 1510 (m), 1530 (m), 1553 (s), 1589 (s), 1614 (m), 2835 (w), 2884 (w), 2901 (w), 2976 (w), 3096 (w), 3146 (w), 3395 (w). Anal. calcd. for $C_{13}H_{13}N_5O_2$ (255.3): C 61.17, H 5.13, N 27.43; Found: C 60.93, H 5.11, N 27.17. ESI HRMS calcd. for $[C_{13}H_{13}N_5O_2+H]^+$: 256.1193; Found: 256.1193.

Supplemental References

1. Rudner J, Lepple-Wienhues A, Budach W, Berschauer J, Friedrich B, Wesselborg S, et al. Wild-type, mitochondrial and ER-restricted Bcl-2 inhibit DNA damage-induced apoptosis but do not affect death receptor-induced apoptosis. J Cell Sci 2001;114:4161-72.
2. Engels IH, Stepczynska A, Stroh C, Lauber K, Berg C, Schwenzer R, et al. Caspase-8/FLICE functions as an executioner caspase in anticancer drug-induced apoptosis. Oncogene 2000;19:4563-73.
3. Brimmell M, Mendiola R, Mangion J, Packham G. BAX frameshift mutations in cell lines derived from human haemopoietic malignancies are associated with resistance to apoptosis and microsatellite instability. Oncogene 1998;16:1803-12.
4. Ierano C, Chakraborty A, Nicolae A, Bahr J, Zhan Z, Pittaluga S, et al. Loss of the proteins Bak and Bax prevents apoptosis mediated by histone deacetylase inhibitors. Cell Cycle 2013;12:2829-38.
5. Soengas MS, Capodieci P, Polsky D, Mora J, Esteller M, Opitz-Araya X, et al. Inactivation of the apoptosis effector Apaf-1 in malignant melanoma. Nature 2001;409:207-11.
6. Drießen D, Biesen L, Müller TJJ. Sequentially catalyzed three-component Masuda-Suzuki-Sonogashira synthesis of fluorescent 2-Alkynyl-4-(7-azaindol-3-yl) pyrimidines: Three palladium-catalyzed processes in a one-pot fashion. Synlett 2021;32:491-6.
