# Supplementary material for: Novel meriolin derivatives activate the mitochondrial apoptosis pathway in the presence of antiapoptotic Bcl-2
Source: Cell Death Discov. 2024 Mar 9;10:125. doi: 10.1038/s41420-024-01901-y (PMC10924942; doi:10.1038/s41420-024-01901-y)

Original Western Blots

(all western blots were scanned via LI-COR Odyssey® imaging system)

Figure 2B
(Ramos)

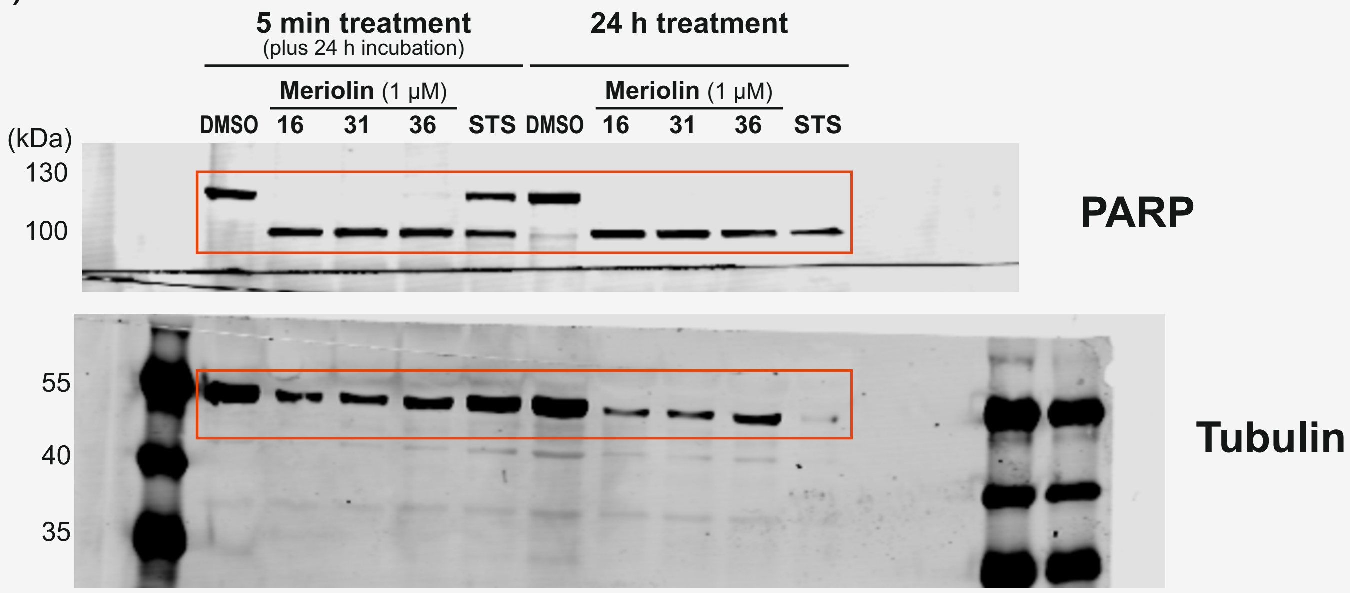

Figure 3G

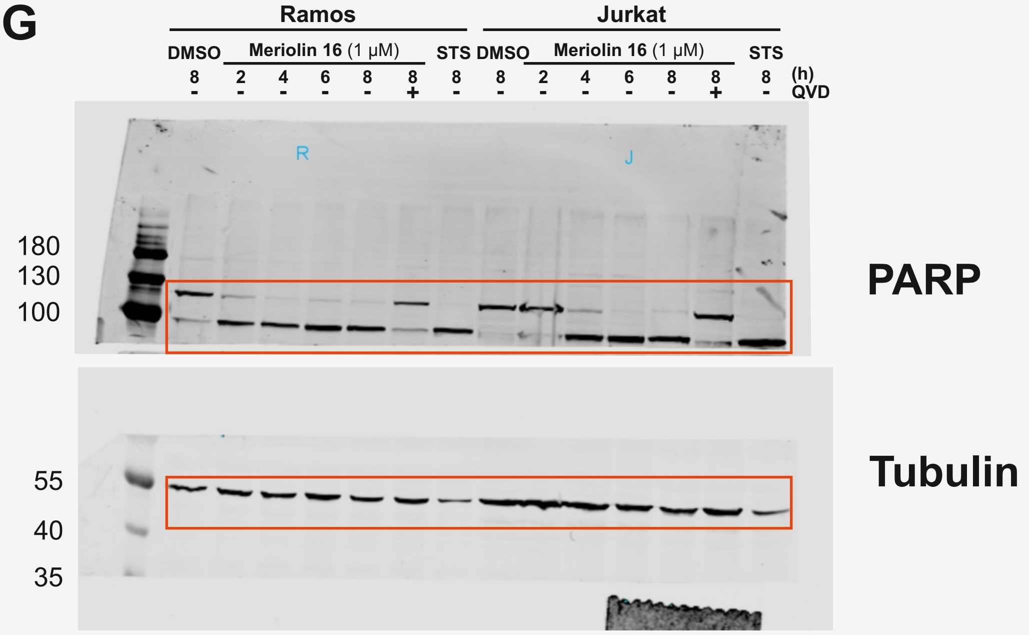

Figure 3H

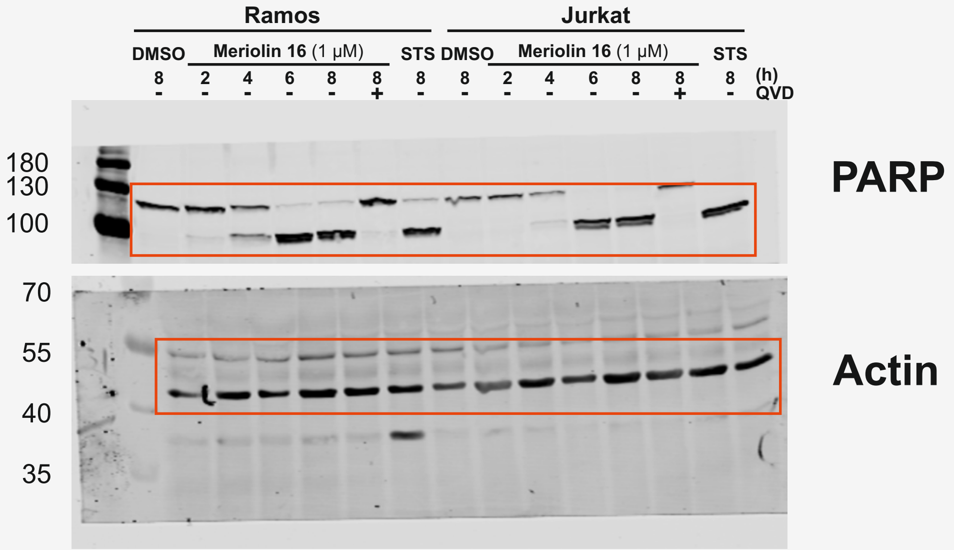

Figure 3I

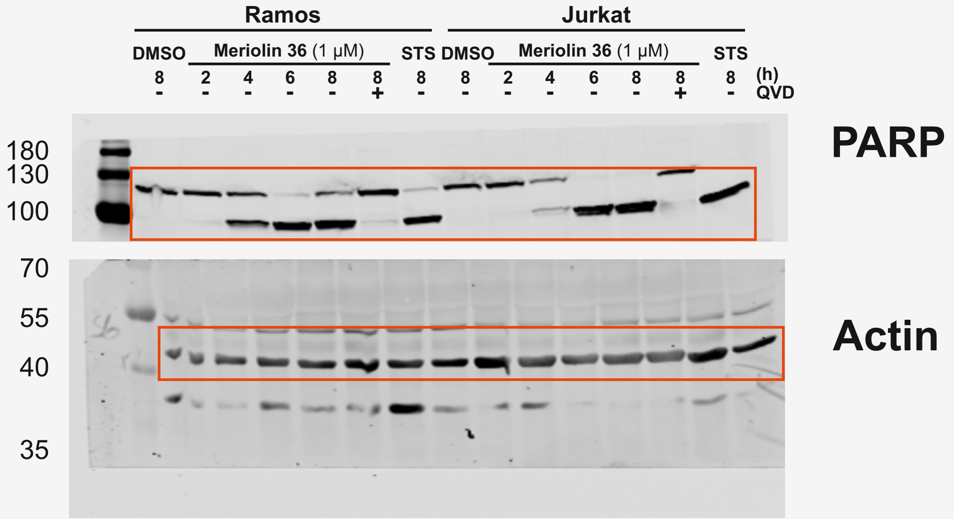

Figure 4B
(left panel)

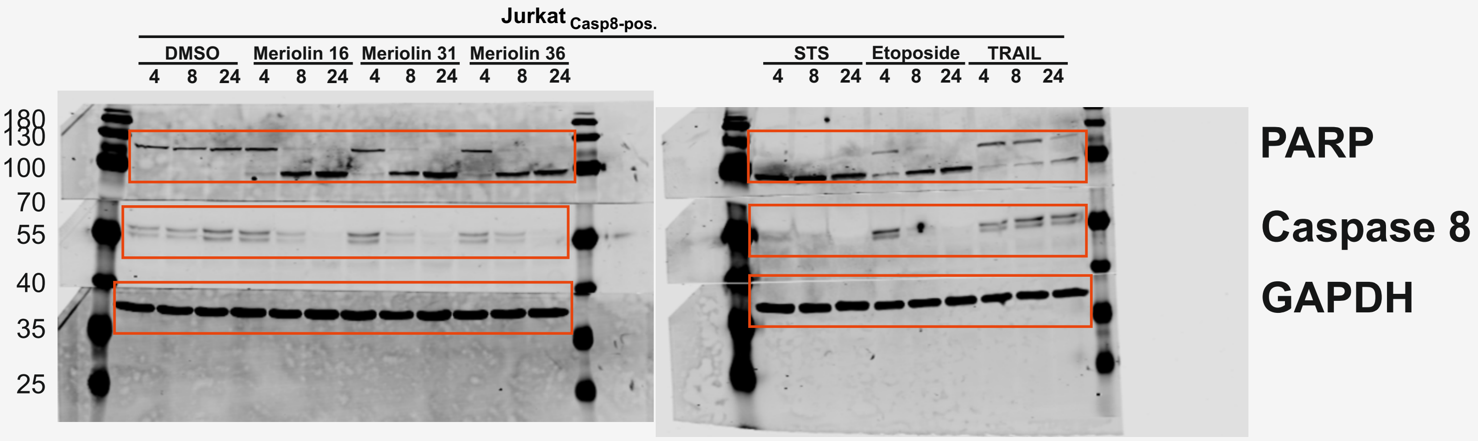

Figure 4B
(right panel)

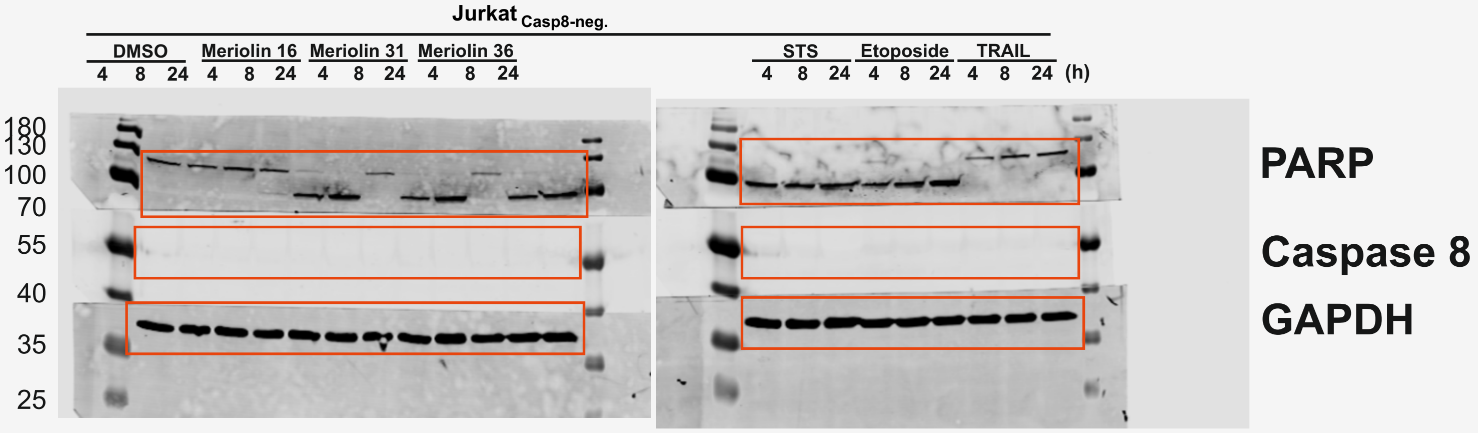

Figure 4D

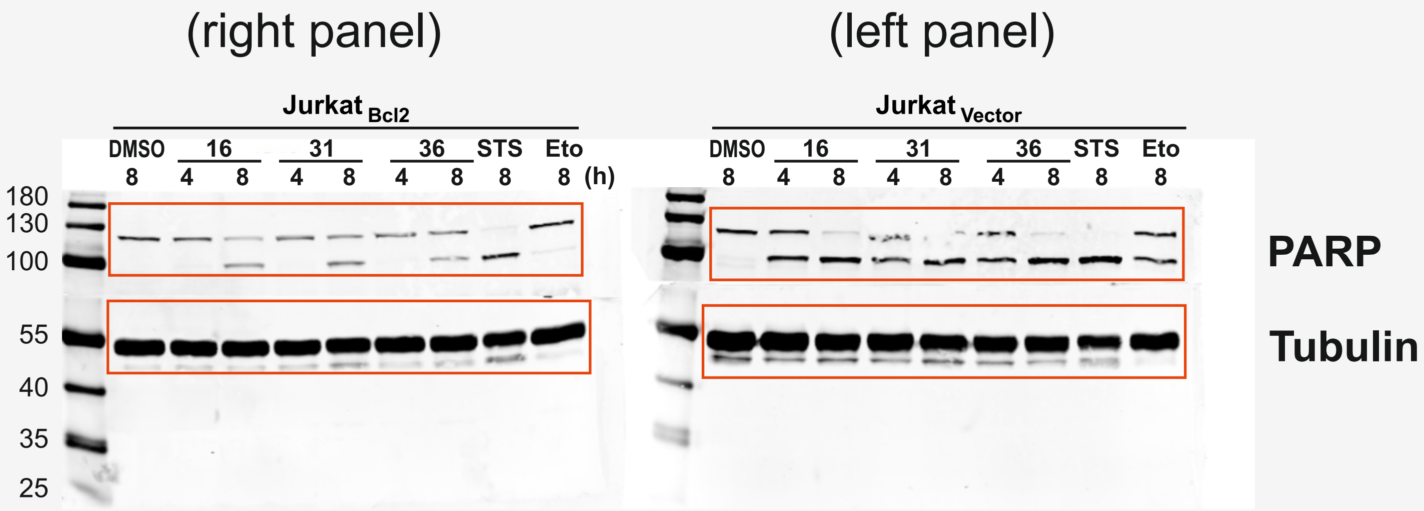

Figure 4F

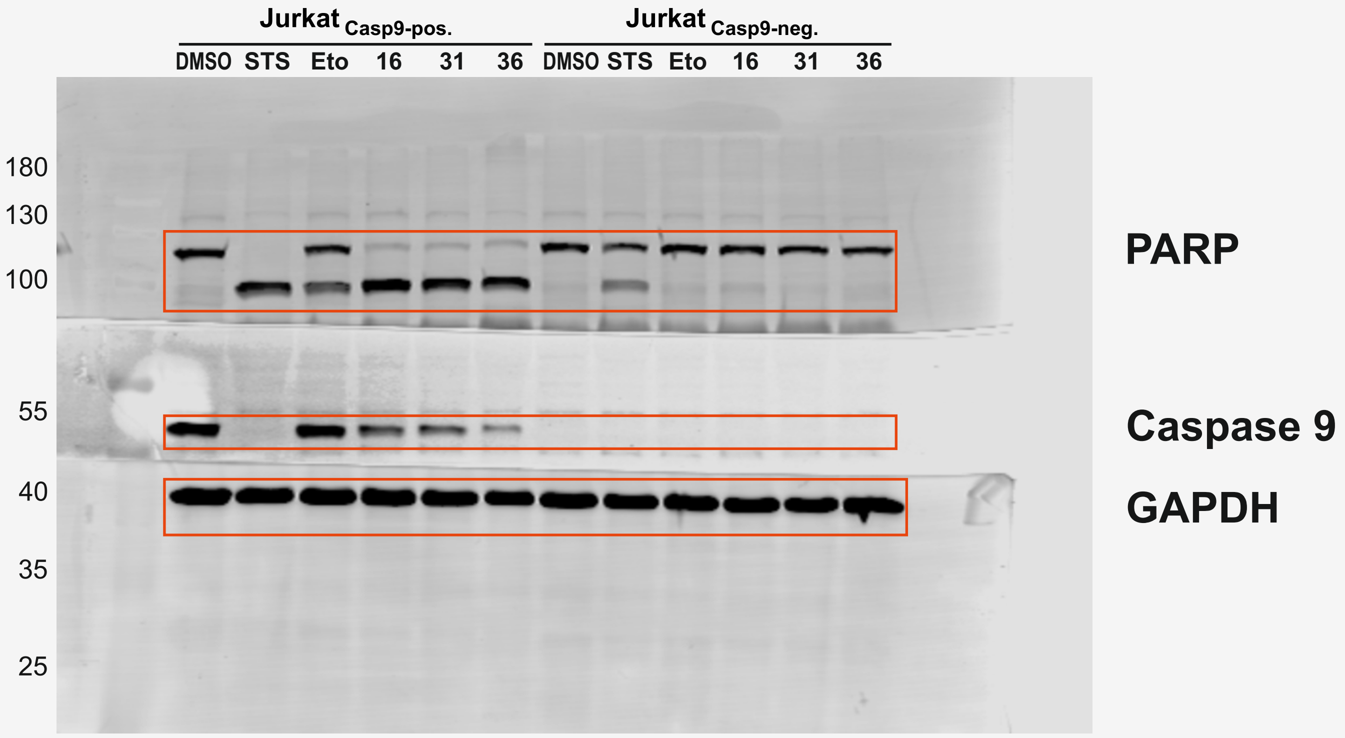

Figure 6B

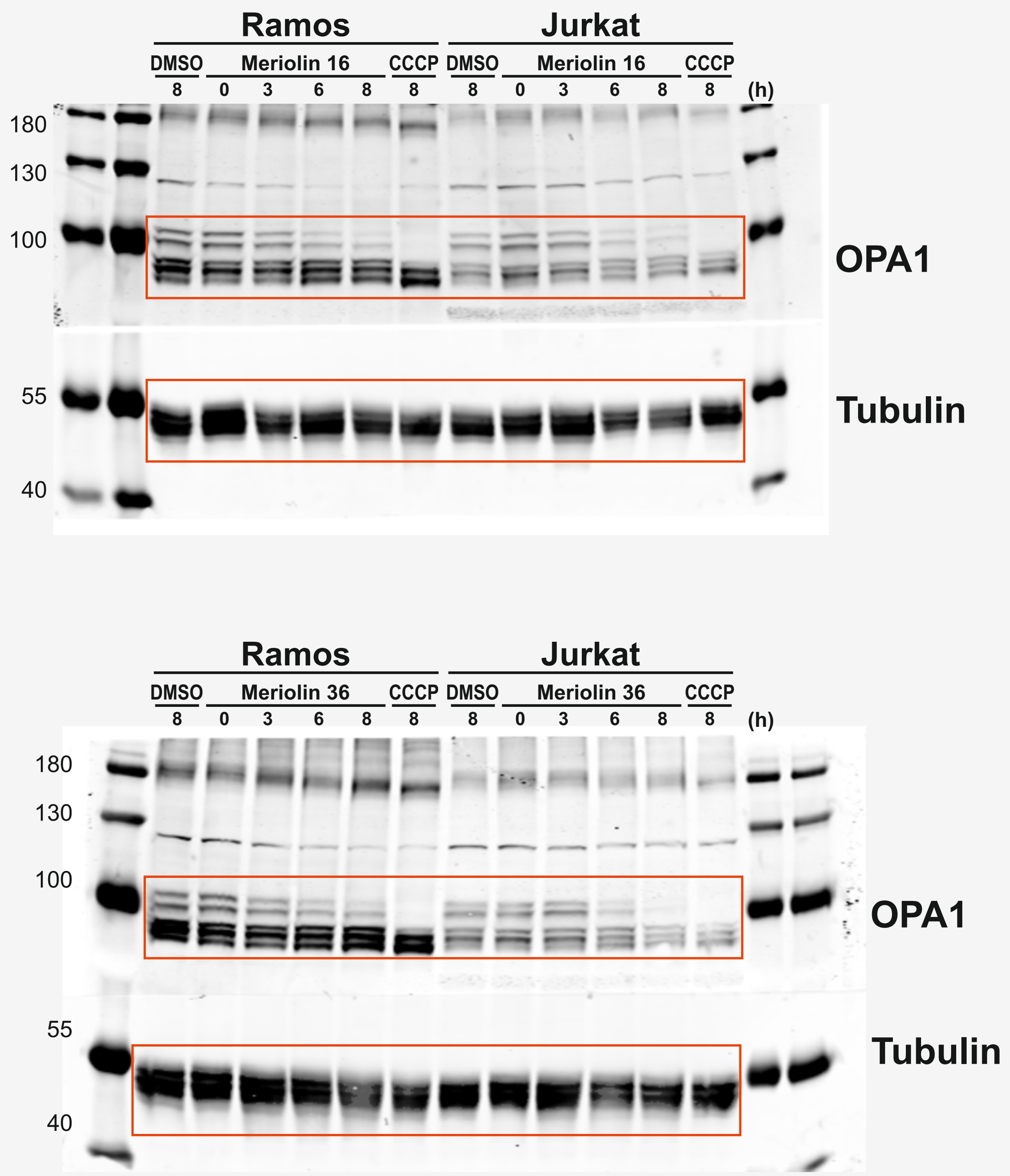

Ramos
Jurkat
DMSO
Meriolin 16
CCCP
DMSO
Meriolin 16
CCCP
8 0 3 6 8 8 8 0 3 6 8 8 (h)
180
130
100
55
40
OPA1
Tubulin
Ramos
Jurkat
DMSO
Meriolin 36
CCCP
DMSO
Meriolin 36
CCCP
8 0 3 6 8 8 8 0 3 6 8 8 (h)
180
130
100
55
40
OPA1
Tubulin

Suppl. Figure 2B

4 h

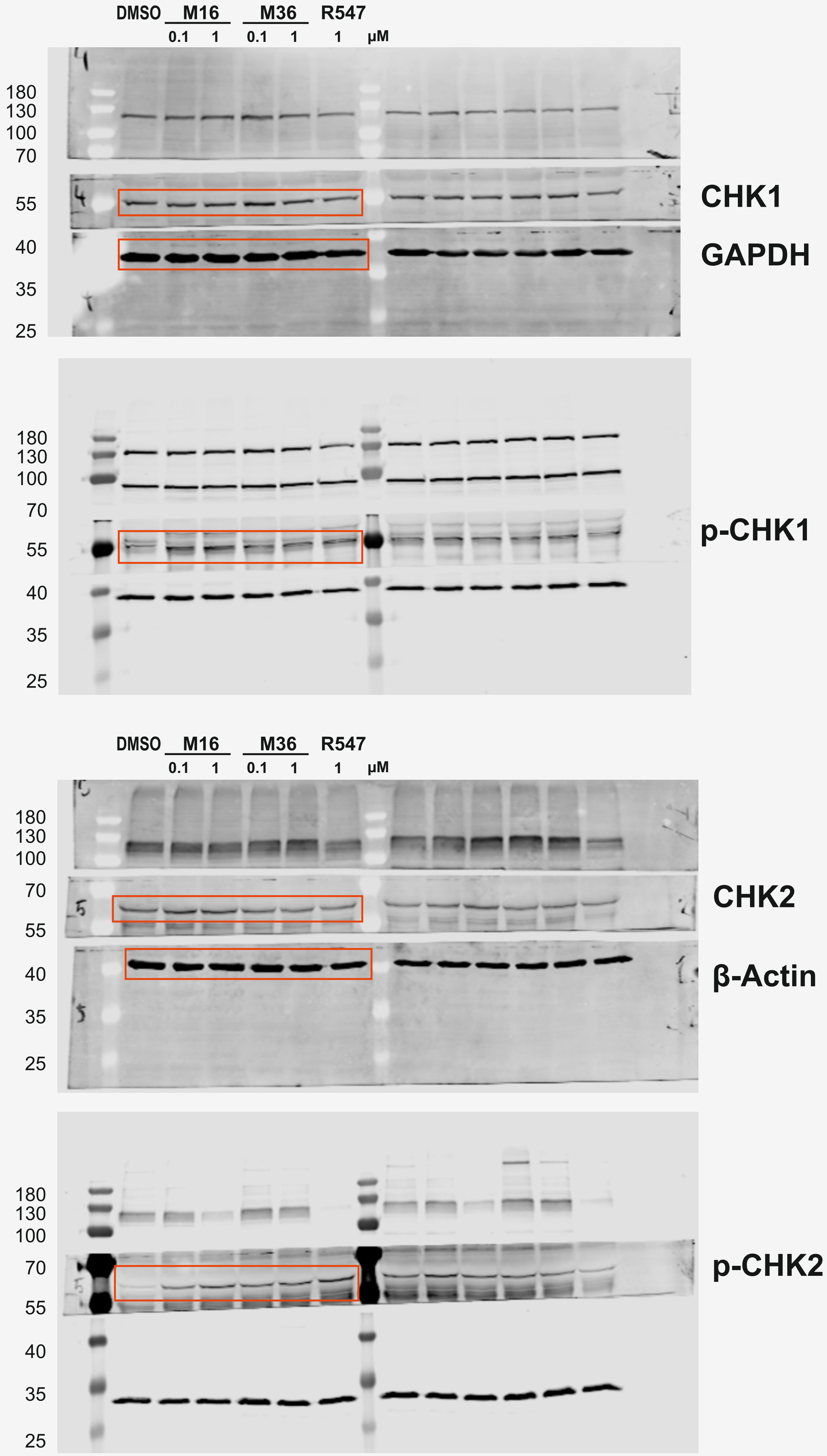

Suppl. Figure 2B

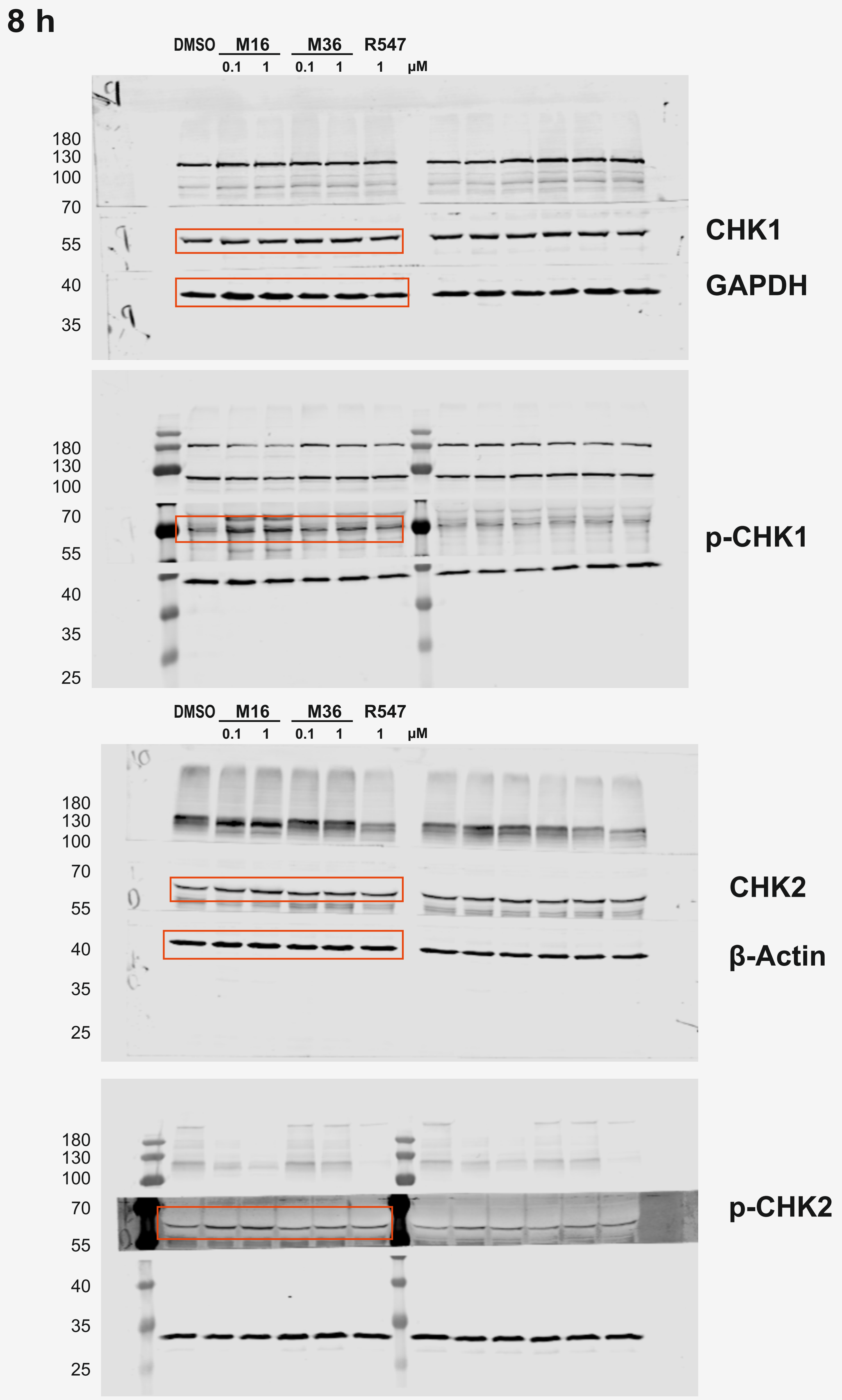

Suppl. Figure 2B

12 h

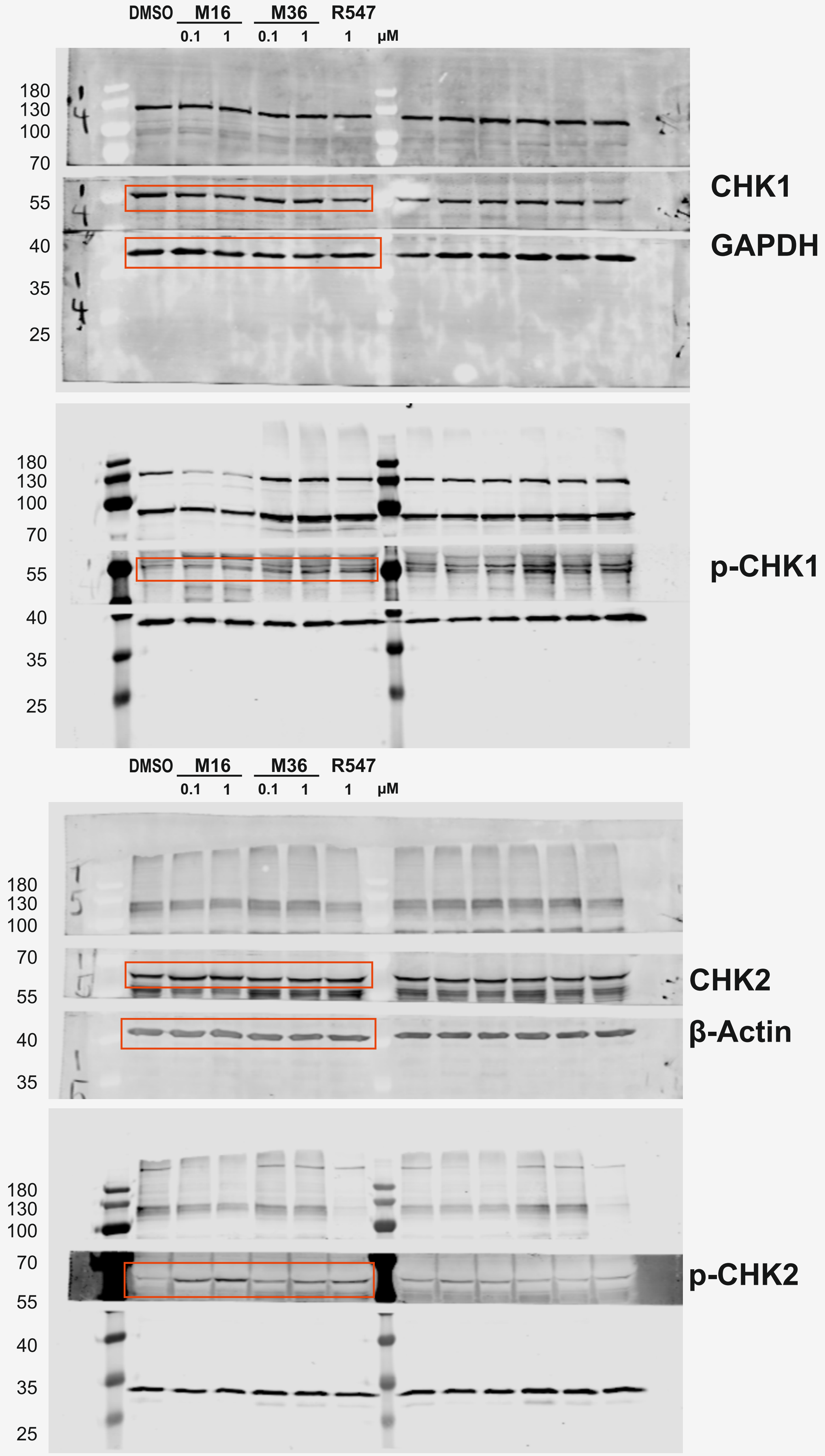

Suppl. Figure 2B

16 h

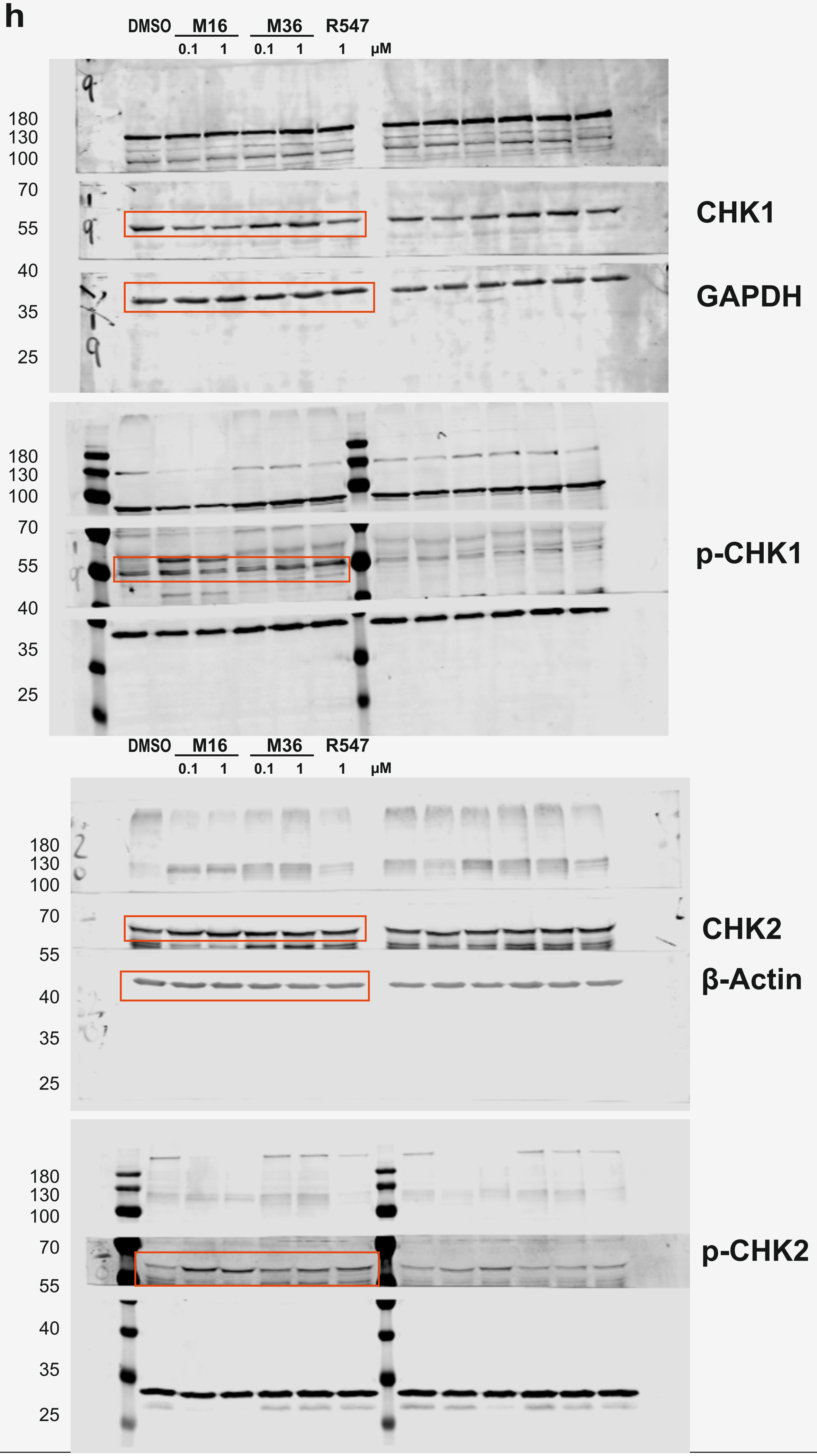

Suppl. Figure 2B

24 h

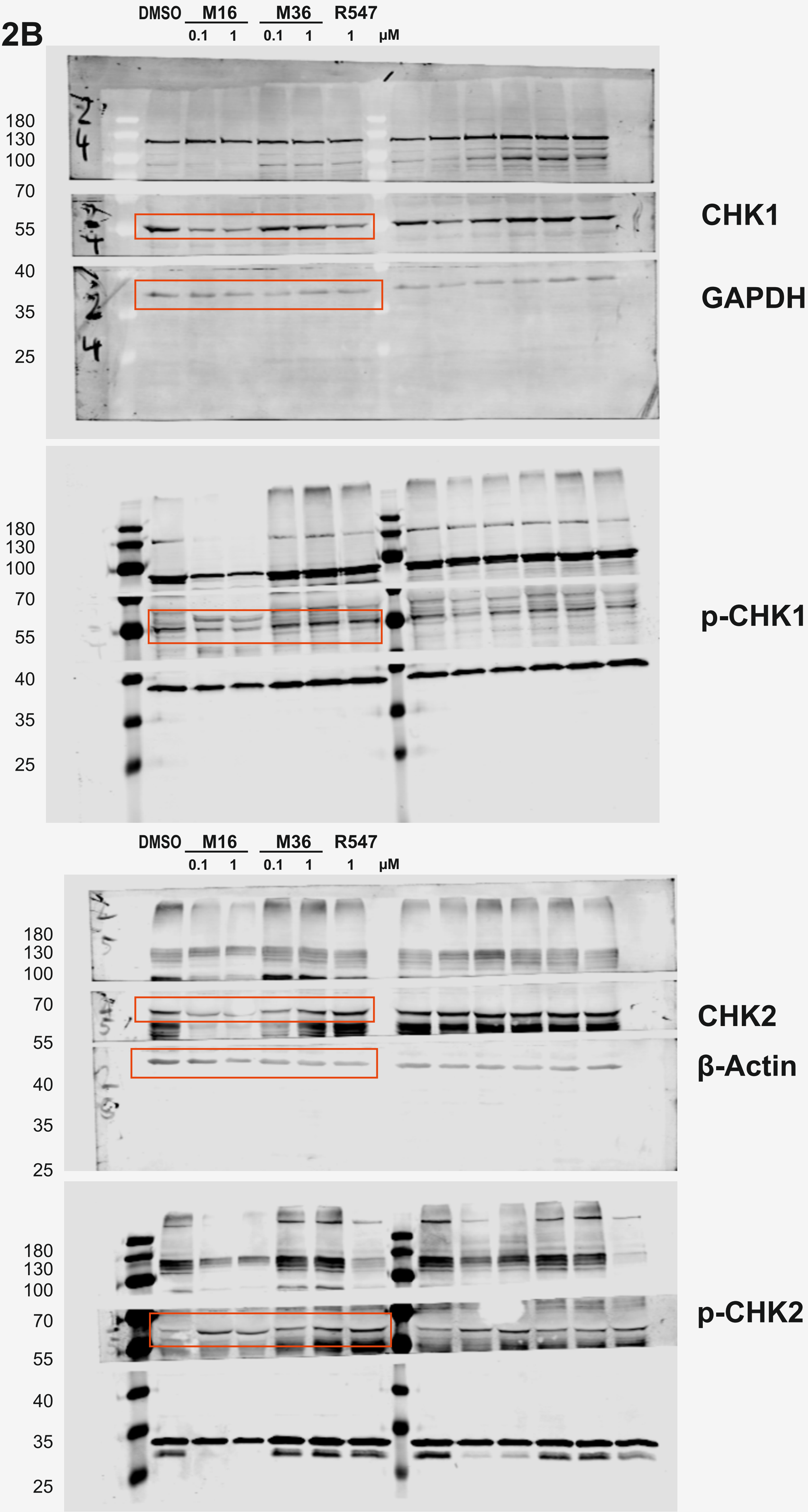

Suppl. Figure 2D

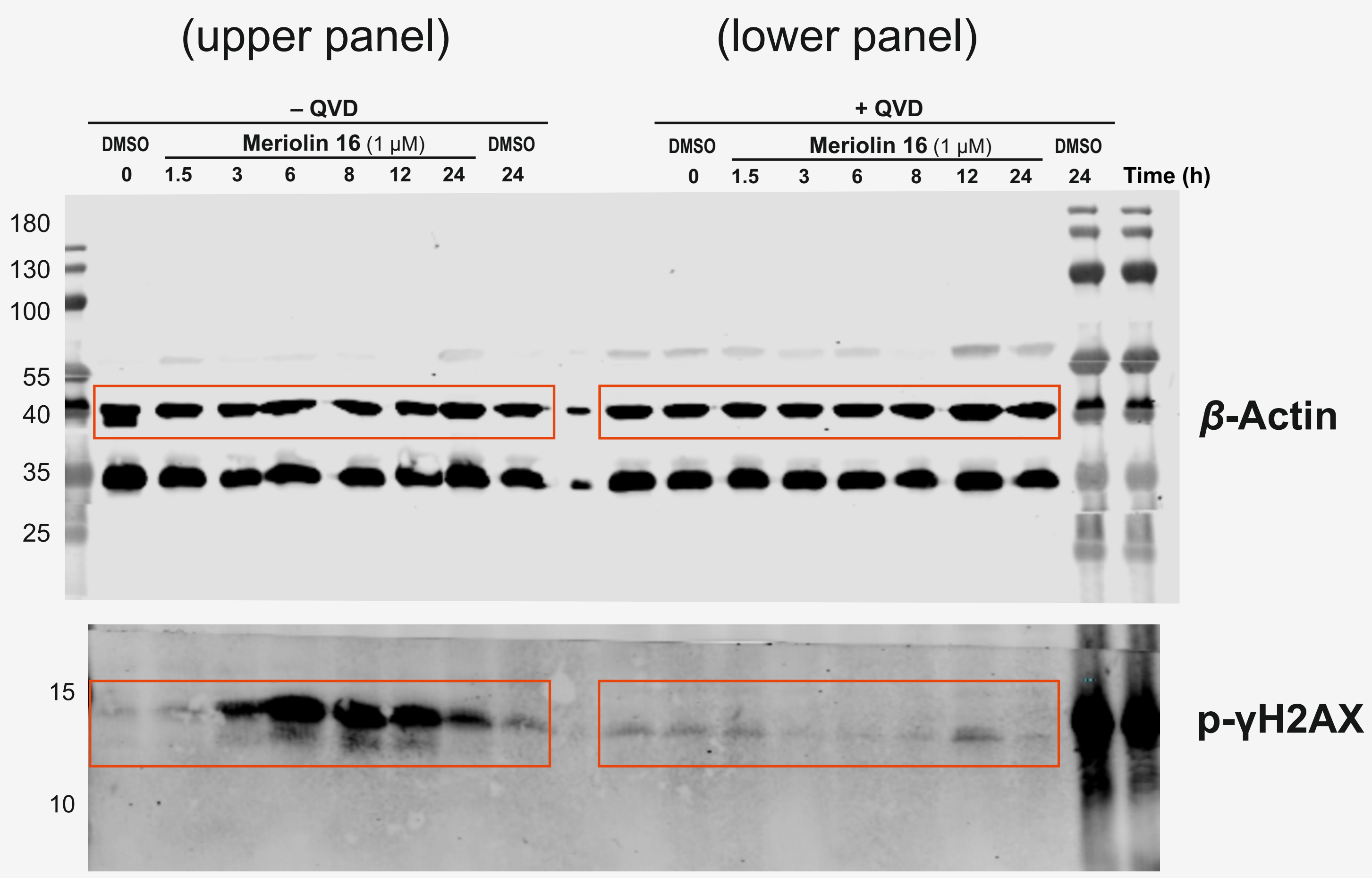

Suppl. Figure 2E

6 h

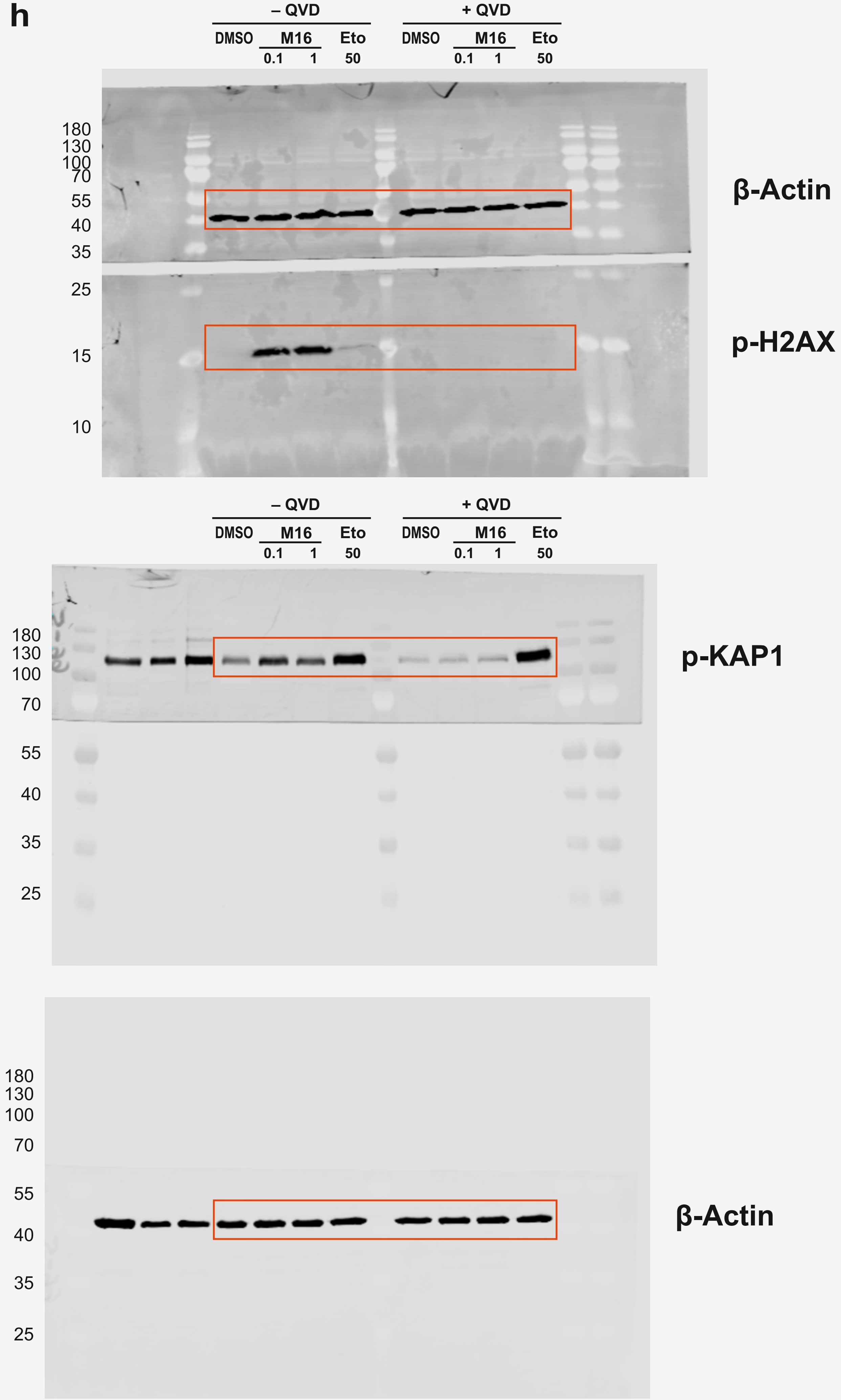

Suppl. Figure 2E

24 h

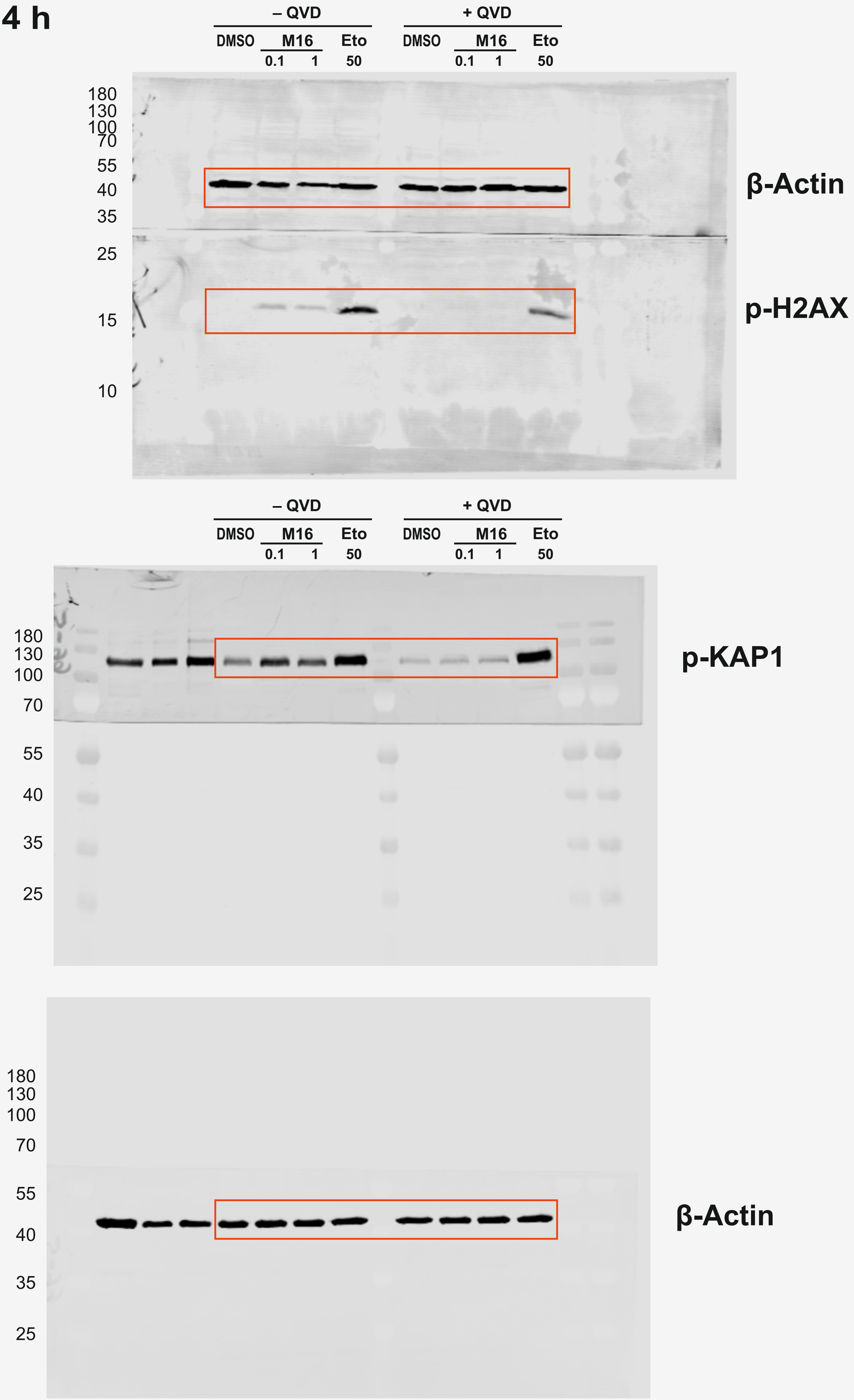

Suppl. Figure 3

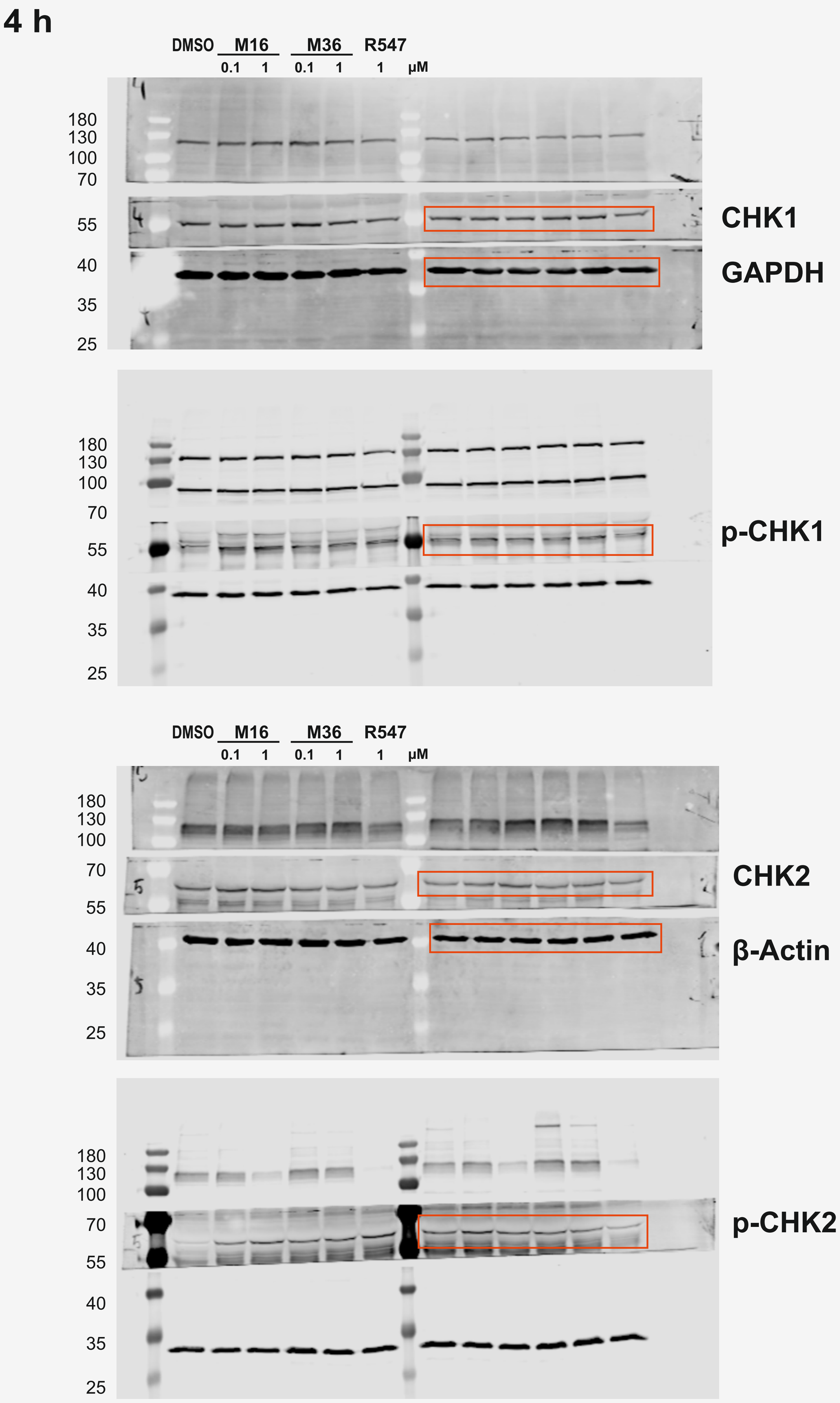

Suppl. Figure 3

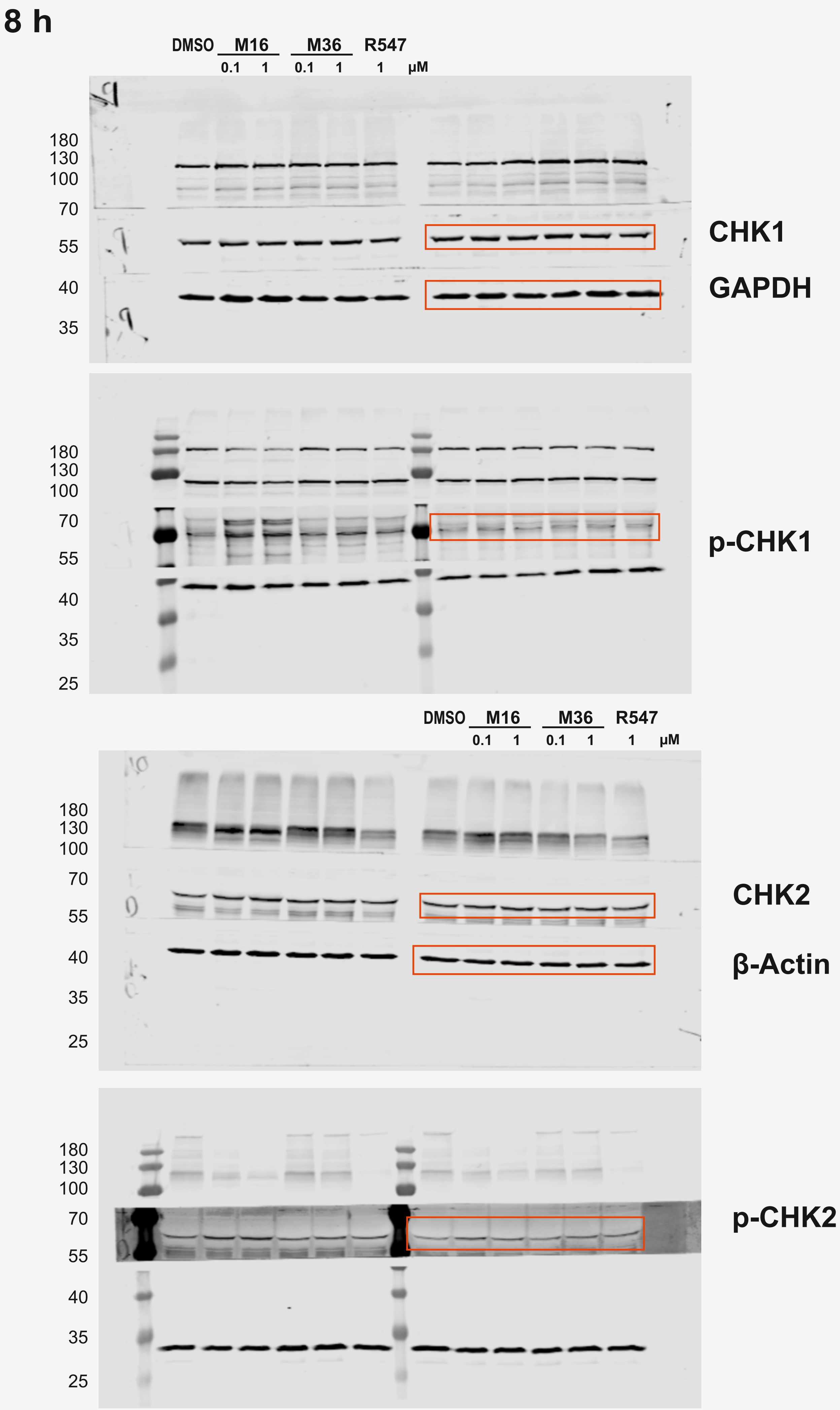

Suppl. Figure 3

12 h

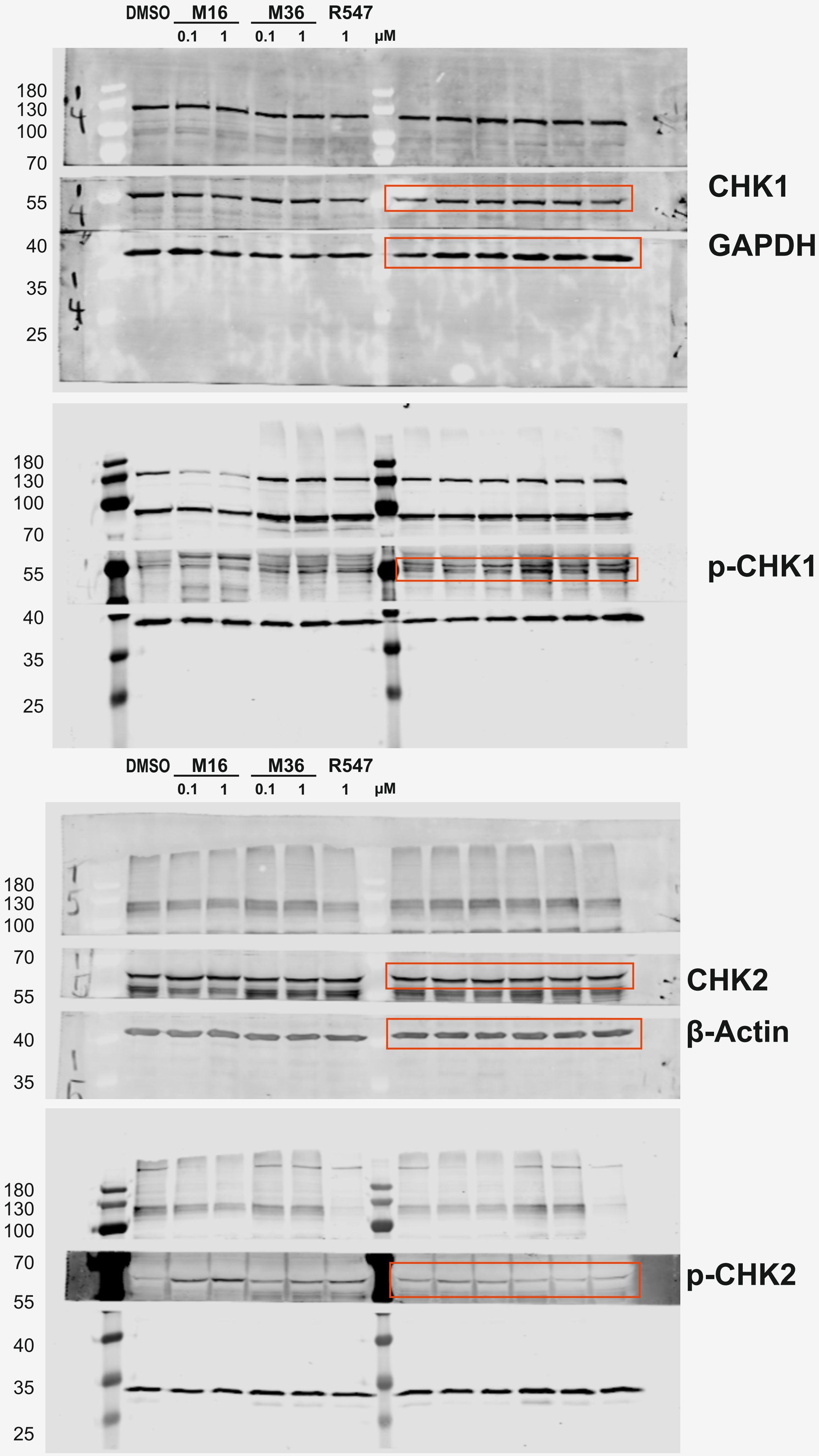

Suppl. Figure 3

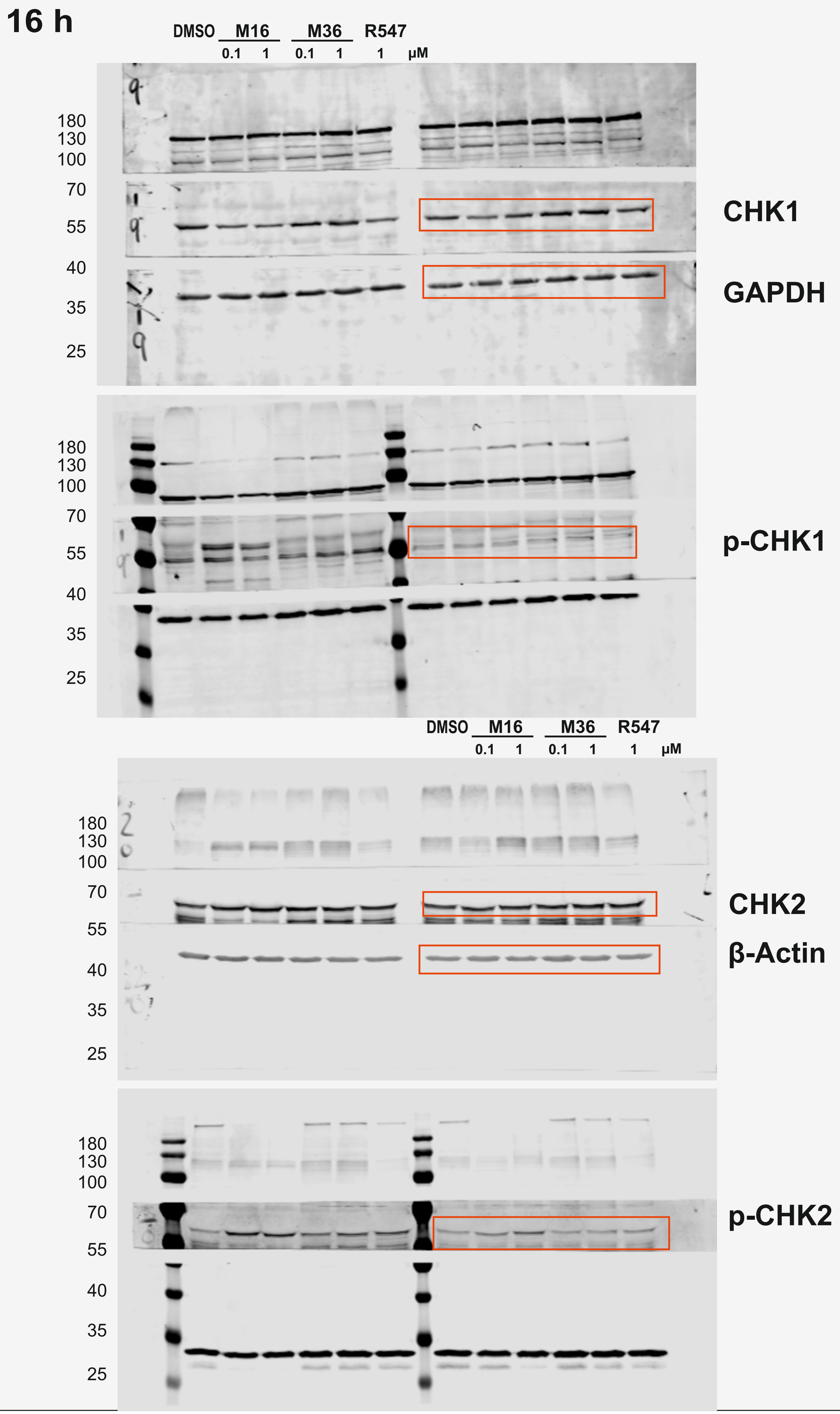

Suppl. Figure 3

24 h

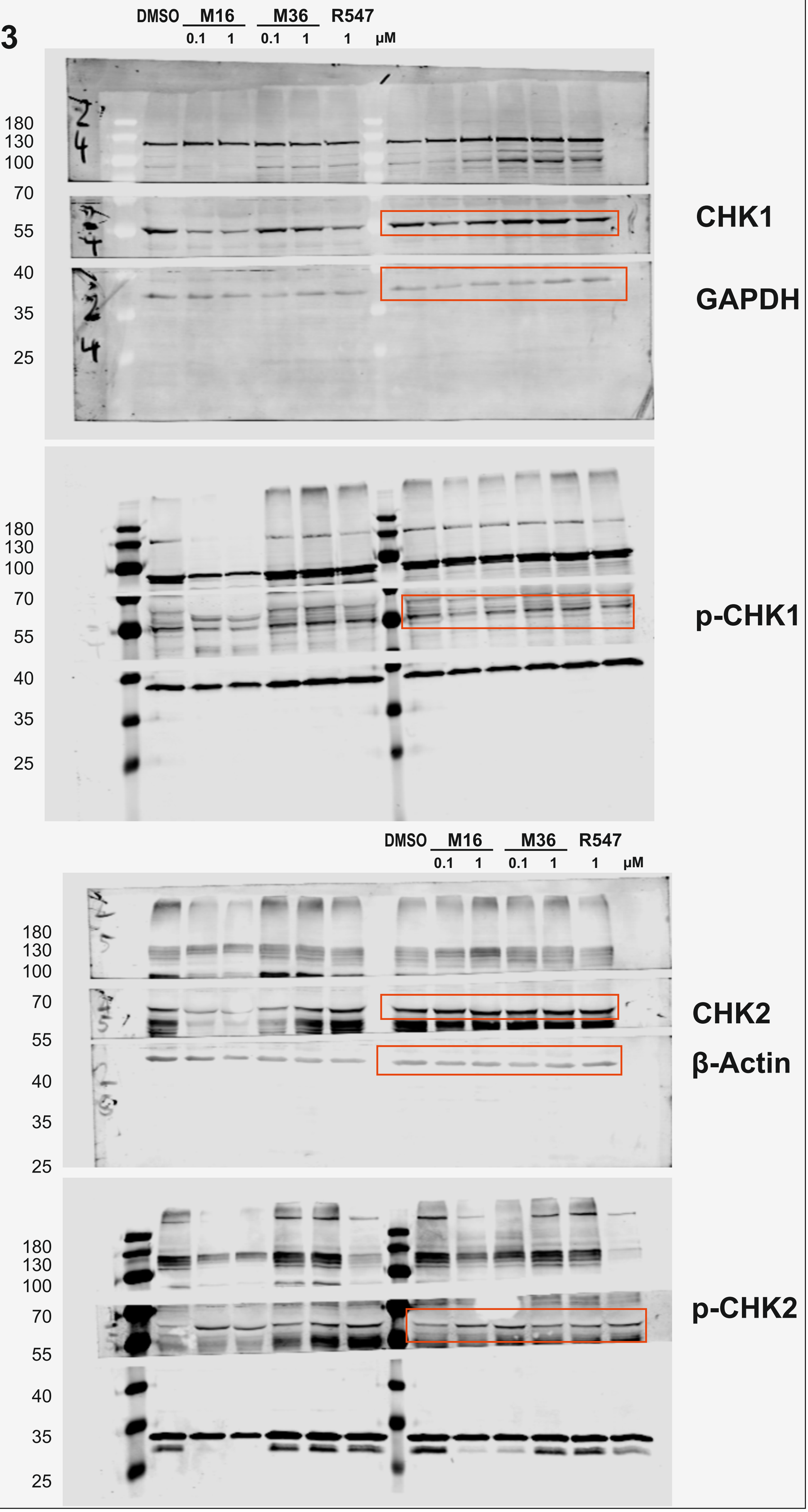

Suppl. Figure 4A,B

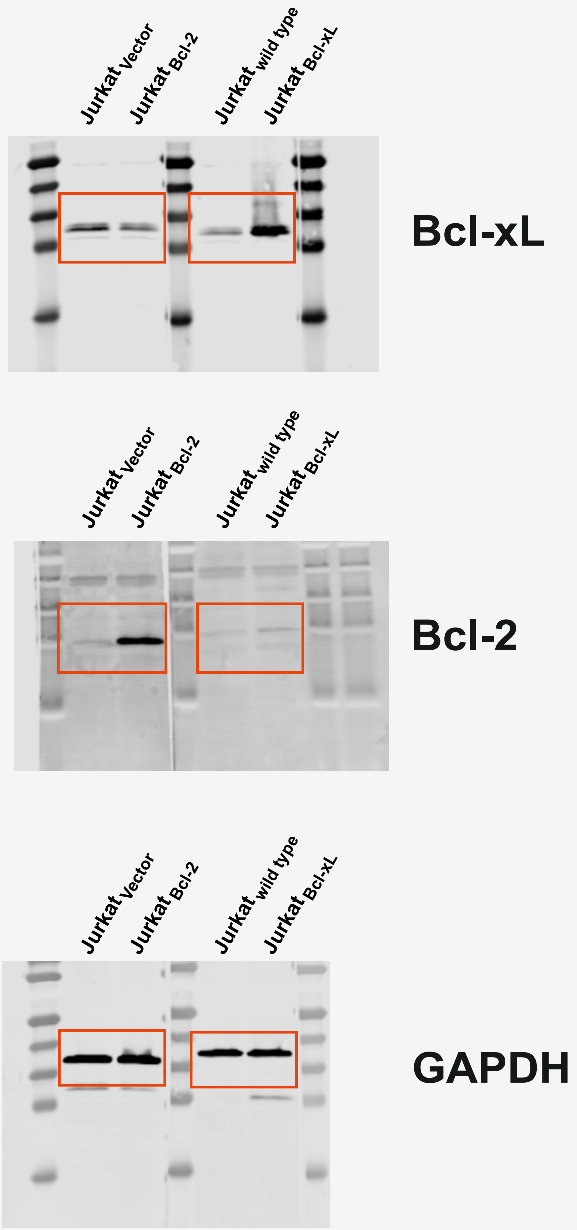

Suppl. Figure 4C

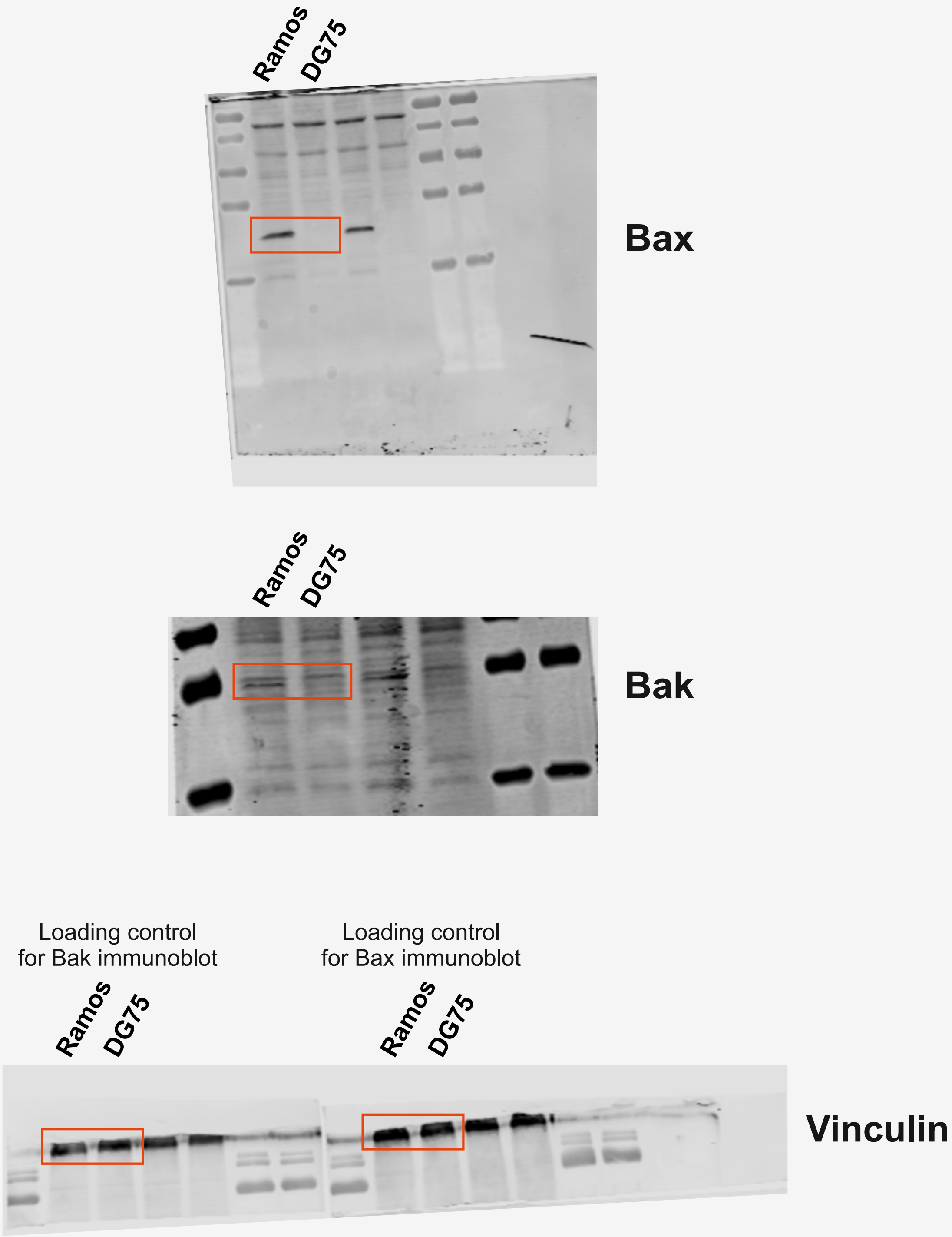

Suppl. Figure 5A

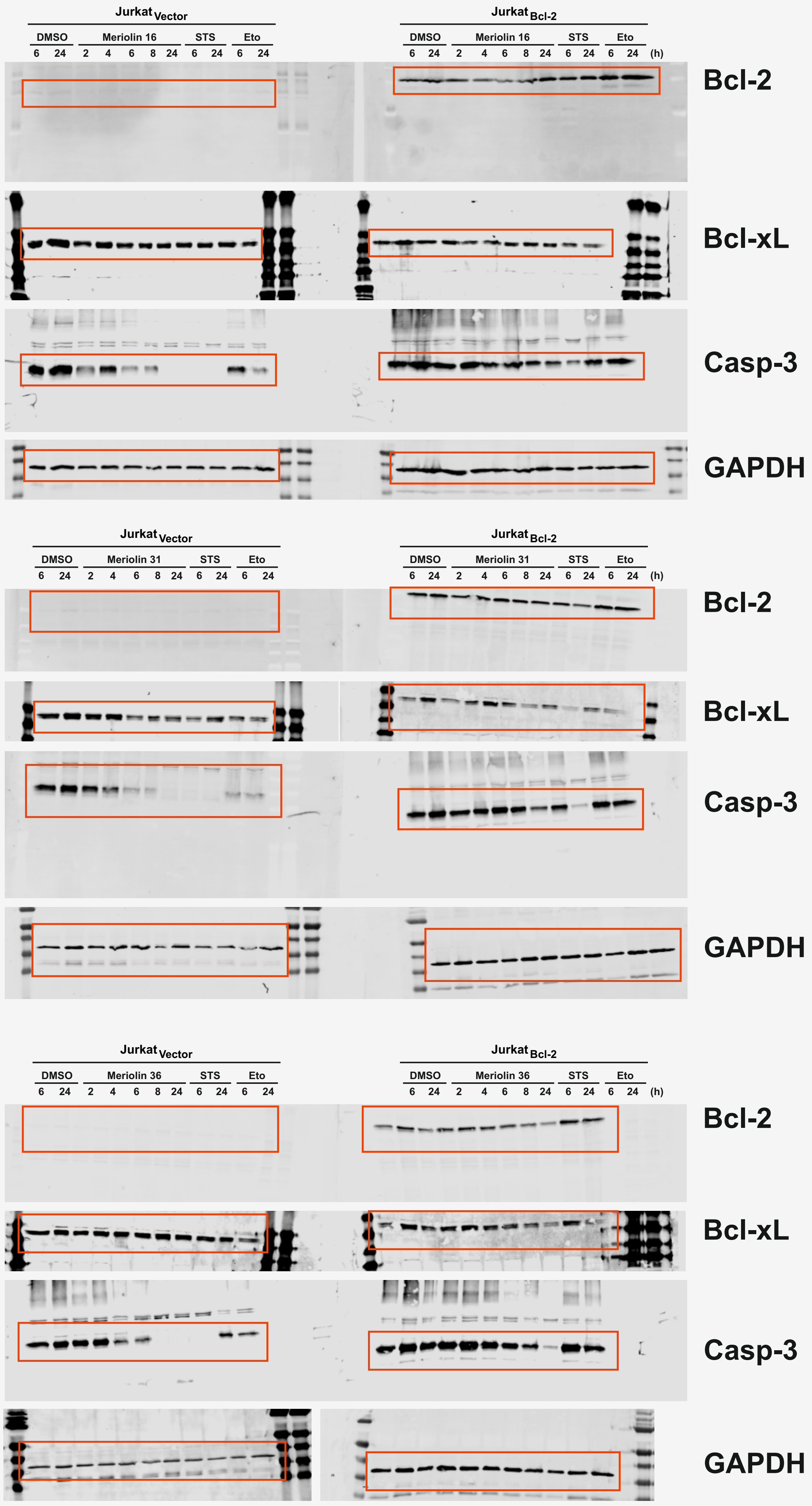

Suppl. Figure 5B

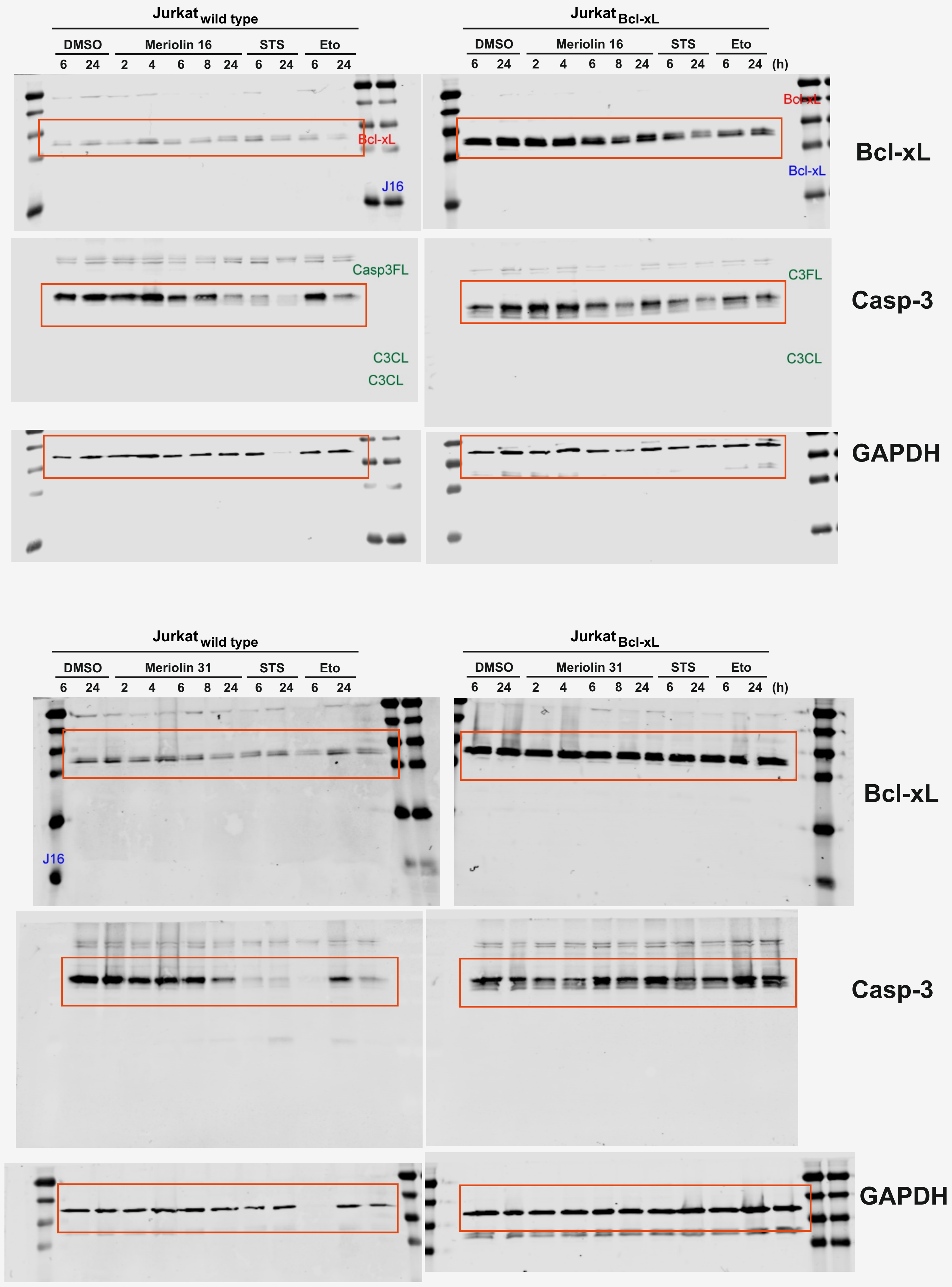

Suppl. Figure 5B

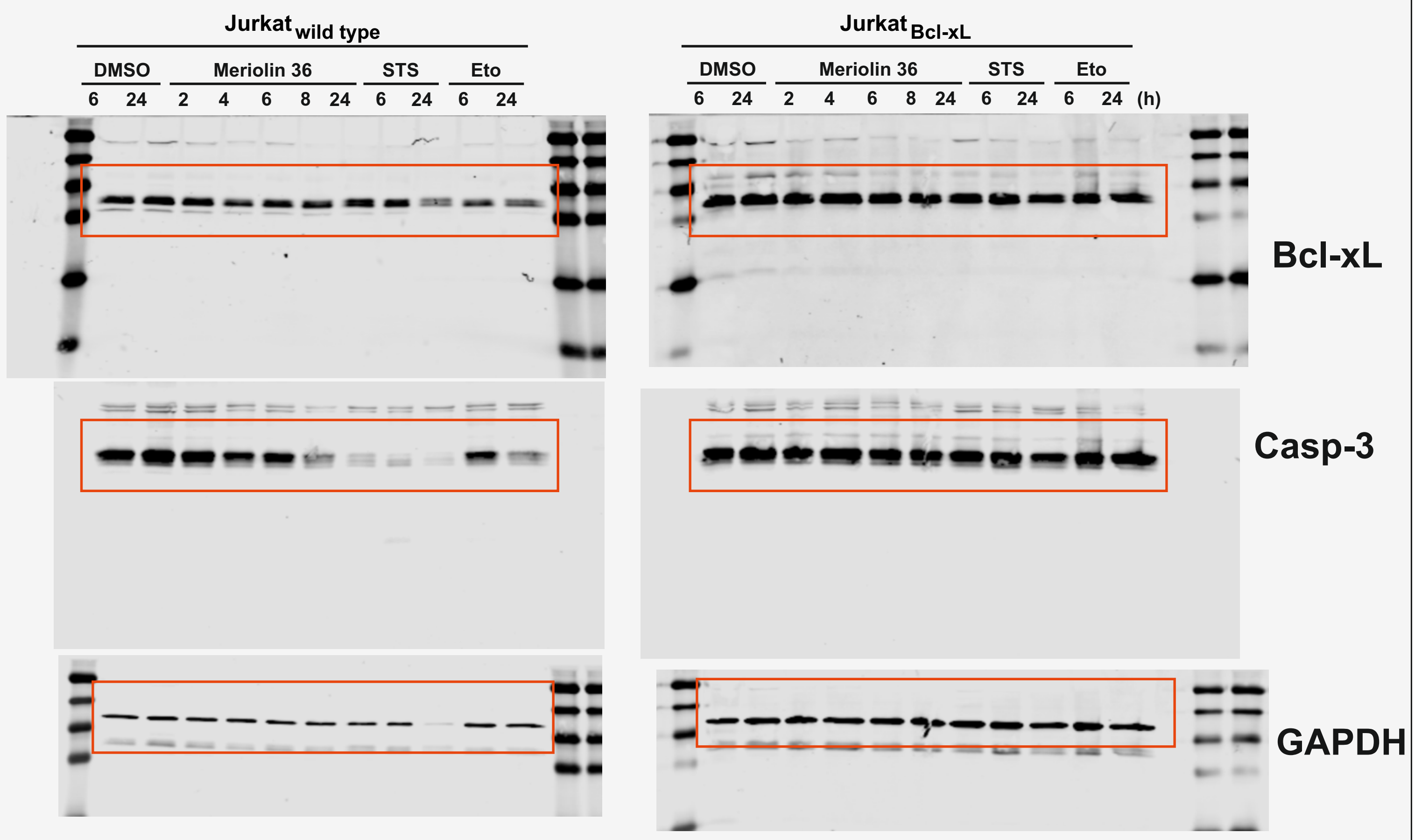

Supplement: Supplementary file 2 — Original Data File [file 41420_2024_1901_MOESM2_ESM.pdf]
